# Overview of Bioactive Fungal Secondary Metabolites: Cytotoxic and Antimicrobial Compounds

**DOI:** 10.3390/antibiotics11111604

**Published:** 2022-11-11

**Authors:** Rafael Conrado, Tainah Colombo Gomes, Gabriella Sales Calaço Roque, Ana Olívia De Souza

**Affiliations:** Development and Innovation Laboratory, Instituto Butantan, Avenida Vital Brasil, 1500, São Paulo 05503-900, SP, Brazil

**Keywords:** fungi, secondary metabolites, cytotoxicity, antimicrobial

## Abstract

Microorganisms are known as important sources of natural compounds that have been studied and applied for different purposes in distinct areas. Specifically, in the pharmaceutical area, fungi have been explored mainly as sources of antibiotics, antiviral, anti-inflammatory, enzyme inhibitors, hypercholesteremic, antineoplastic/antitumor, immunomodulators, and immunosuppressants agents. However, historically, the high demand for new antimicrobial and antitumor agents has not been sufficiently attended by the drug discovery process, highlighting the relevance of intensifying studies to reach sustainable employment of the huge world biodiversity, including the microorganisms. Therefore, this review describes the main approaches and tools applied in the search for bioactive secondary metabolites, as well as presents several examples of compounds produced by different fungi species with proven pharmacological effects and additional examples of fungal cytotoxic and antimicrobial molecules. The review does not cover all fungal secondary metabolites already described; however, it presents some reports that can be useful at any phase of the drug discovery process, mainly for pharmaceutical applications.

## 1. Introduction

Natural products can be obtained from plants, animals, microorganisms, or marine organisms, which are present in different environments, such as in the aquatic, terrestrial, or even in the air. Natural products have been relevant sources for drug discovery and the development of medicines since ancient times [1,2,3].

Newman and Cragg [1] showed that from January 1981 to September 2019, at least 1881 new drugs were approved worldwide for the treatment of all types of diseases, and among them, 75.38% (1418) were derived from natural products.

**Figure 1:**
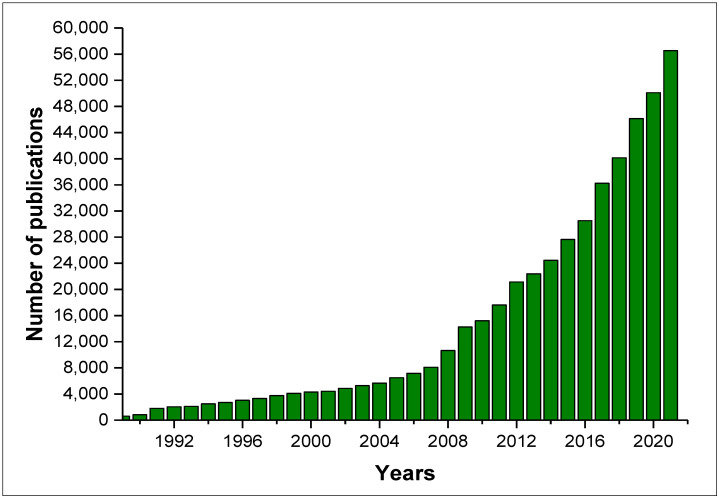
Overview of publications profile containing in their topic the keyword “natural product”, from 1989 to 2021. Source: Web of Science database (accessed on 15 August 2022).

According to the Web of Science, from January 1989 to August 2022 (Figure 1), there were 486,109 publications related to the keyword “natural products”, 14,364 related to “natural products fungi”, and 9615 addressing “natural products secondary metabolites”. Figure 1 shows the huge increase in publications related to natural products since 1989, and in addition, an increase of approximately 4-fold in the number of publications related to “secondary metabolite natural products” in the last 10 years can be observed.

Secondary metabolites (SMs), also known as natural products, are biomolecules with a low molecular weight that, although not essential for the development of its producer, can increase their tolerance to different types of environmental stresses and hostile conditions, and consequently, their survival rate [1,2,3].

Bioactive SMs are produced mainly by microorganisms, which can be isolated from soil microbiota, marine environments, or extreme environments. The most promising are those obtained from underexplored niches, including plant-associated microbes called endophytes, which live in symbiotic relationships with host plants in their internal tissues and structures. Endophytes can be isolated from herbaceous plants, grasses, trees, shrubs, mosses, ferns, and aquatic plants [4].

Recent research has demonstrated that among fungi species, the endophytes are a great source of bioactive SMs, and approximately 20,000 compounds were already isolated from these microorganisms [5,6,7,8,9,10]. The diversity of fungal endophytes is wide mainly due to the different environments to which they are exposed, also considering the seasonal variations, host plants’ genotype, and physiology [11]. These microorganisms have functional diversity and, in association with plants, can produce SMs that protect the host from insects or other microorganisms’ attacks [12].

SMs are used by microorganisms as chemical signs for communication, to defend the habitat, or to inhibit the growth of competitors [13] and differ from their primary metabolite equivalents by the timing of their biosynthesis and dispensability for fungal growth [14]. The evolution of SMs biosynthesis by microorganisms is mainly due to their exposure to complex ecosystems.

Some SMs have afforded society-changing benefits; however, some compounds are associated with serious problems and negative impacts, such as mycotoxins, due to their widespread occurrence as food contaminants for humans and livestock, as well as mold-contaminated indoor environments [15]. Even so, previous data shows that among the 500,000 SMs described, around 70,000 compounds are obtained from microorganisms, and roughly 33,500 are bioactive compounds, of which 47% are of fungal origin (15,600) [16,17].

## 2. Obtaining Microorganism Strains for Biosynthesis of Bioactive Secondary Metabolites

**Figure 2:**
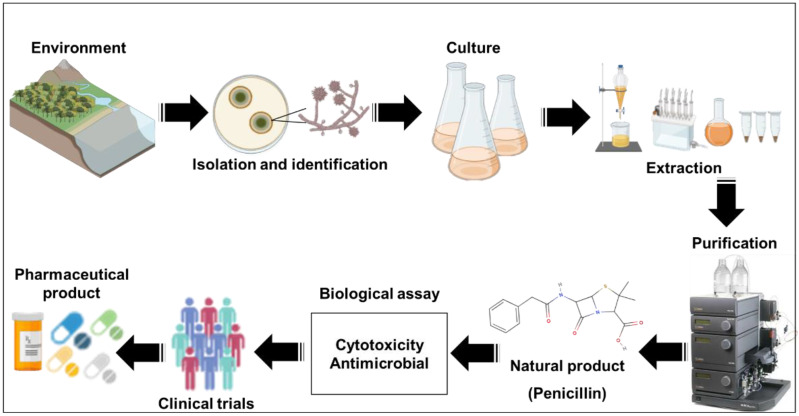
Representation of fungal strain isolation and development of medicines containing natural products as the bioactive component. Penicillin is being used just as an example.

Figure 2 briefly illustrates a few steps from the isolation of a fungi strain to the development of a pharmaceutical product. The process begins with the isolation of the fungus strain from diverse environmental sources (e.g., water, plants, and soil), and the culture of the microorganisms. The product of the culture is processed for extraction and purification of the SM. Subsequently, biological assays and clinical trials are performed to define the possibility of future pharmaceutical applications of the SM (Figure 2).

The limitations for the discovery of new SMs from microorganisms begin in the handling and isolation of new strains. The isolation process of microorganisms, including that for endophytes, is well described; however, there is some challenge in isolating new fungi species, mainly due to the current techniques applied for the cultivation and strains purification that usually have remained the same in the last years. For obtaining new species, it is mandatory to design new approaches that are able to simulate the condition of the natural habitat from which the microorganisms were isolated. In this aspect, it is essential to overcome challenges related to growing conditions such as pH, temperature, nutrients, and preservation in the laboratory.

## 3. Obtaining Fungal Secondary Metabolites and Structural Characterization

Once the limitations for microorganisms isolation have been overcome, the next step is to proceed with its culture, followed by extraction, purification, and physicochemical characterization of the SMs. These steps are very well described and widely applied in natural product research.

Fungal culture for SMs biosynthesis can be performed in either solid-state fermentation (SSF) or submerged fermentation (SmF). The first one, on a solid substrate, can produce a high concentration of metabolites. In this system, the low water concentration favors oxygen transfer for the growth of the microorganisms. Among solid substrates, rice straw, rice hull, and sugarcane bagasse are common [18]. The SmF occurs in aqueous liquid nutrient media, and it is the most widely used due to the ease of control of fermentation parameters such as pH, temperature, dissolved oxygen, and types of culture media [18].

A good example is the lovastatin production that has been attempted in both SSF and SmF, where using SmF, the lovastatin production is about five times higher [19].

In addition to different strategies for the microorganism culture, the profile of the obtained metabolites also depends on the extraction technique and solvent applied for it. Appropriate organic solvents such as chloroform, dichloromethane, hexane, or ethyl acetate are the most commonly applied for the extraction of the metabolites from both culture methods. However, it is important to optimize the process to obtain a full profile of SMs. The most applied techniques are liquid–liquid extraction (LLE) and ultrasonic-assisted extraction (UAE). Through the LLE, the metabolites from the aqueous phase are transferred to the organic solvents and obtained by phase separation; in the UAE, ultrasound waves increase the extraction efficiency by disrupting cells for the effective release of the compounds to the solvents [20].

For SMs extraction, an effective and recent innovative approach is the Solid-Phase Extraction Embedded Dialysis (SPEED technology), which is based on a physical separation between the microbial biomass and the resin used for in situ solid-phase extraction [21]. This technology consists of a two-layer barrier with an external nylon filter cloth (NFC) and an internal dialysis tube (DT) containing the resin beads. The molecular cut of the tube allows only molecules with appropriate molecular weight can flow from the culture broth to the resin. This selectivity makes SPEED technology a very interesting tool for the study of specialized microbial metabolites.

In addition to the development of genome-based technologies, the employment of modern omics tools and dereplication strategies can potentially increase the efficiency of the discovery of new molecules from microorganisms making the process approach wider and more effective [22]. To avoid the rediscovery of known molecules, it is important to identify the obtained compounds and to prioritize those potential strains for new compound biosynthesis. In this regard, the dereplication using a metabolomics profile based on mass spectrometry has been useful in selecting these strains, and, in addition to that, the data can be integrated into a Global Natural Product Social Molecular Networking (GNPS) [23]. Nuclear magnetic resonance (^1^H, ^13^C NMR) spectroscopy [24,25], 2D NMR [26], and mass spectrometry (MS, MS/MS) [27,28] fingerprints are widely used, and well-established techniques for the dereplication of natural products, and in addition to that, Fourier transform infrared spectroscopy (FTIR) was proposed by Grkovic et al., [29] to be equally valuable in the dereplication workflow. FTIR application is supported by modern instruments, which have a relatively small footprint, are sensitive, and provide a large data set across the spectroscopic range, suitable for library generation and pattern matching [29].

The dereplication combining different analytical tools enables rapid identification of known compounds and introduces knowledge of active principle structural class early in the isolation workflow [24].

After the extraction, the fractionating and purification of compounds present in a crude extract are performed mainly by chromatographic techniques, including thin layer chromatography (TLC), liquid chromatography (LC), high-performance liquid chromatography (HPLC) [30,31,32], and supercritical fluid chromatography (SFC). The HPLC analyses can be operated by distinct modes categorized mainly by the type of stationary phase, being (i) normal phase, (ii) reversed phase (RP-HPLC), (iii) hydrophilic interaction liquid chromatography (HILIC), and (iv) ion chromatography [32]. The interaction of the sample with the stationary phase depends on the relative solubilities, surface absorption or adsorption, ion exchange, and steric effects [32].

Normal-phase HPLC and RP-HPLC differ from each other according to the stationary and mobile phases used. In normal HPLC, the separation of non-polar compounds occurs at the stationary phase that is composed of hydrophilic groups, followed by the polar ones in the sequence. In RP-HPLC, lipophilic groups form the stationary phase, and the polar compounds are eluted first [30,31,32].

RP-HPLC has a wide capacity for analyzing different compounds due to its broad applicability and reproducibility. The hydrophobic properties of the stationary phase allow the reverse HPLC use for separation of mixtures of hydrophilic (i.e., polar), hydrophobic (i.e., non-polar), ionic and/or ionizable molecules resulting in the purification of their components, according to the employed procedure [31]. The chromatographic selectivity of RP-HPLC can be changed through modifications in the mobile phase characteristics, and excellent resolution can be achieved, even for very closely related structures present in the mixture.

The HPLC, ultra-performance liquid chromatography (UPLC), and ultra-high PLC (UHPLC) can be temperature controlled and are usually coupled with different detectors. The analytes can be detected by UV/Vis, photodiode array (PDA), and/or mass spectrometry (MS) [32]. The UV/Vis detector can be operated in dual wavelength mode, whereas the PDA can measure the peaks in a wavelength-scanning mode resulting in a total absorbance spectrum of a single peak. The main advantages of PDA detection are the resolution and wavelength range allowing the selection of the best wavelength for analysis. In addition to that, for specific naturally fluorescent compounds, the detection can be performed by fluorescence (FLD). Moreover, although not regularly used, the chromatographs can be coupled with detectors system through electrical conductivity, electrochemical, refractive index (RI), light scattering, evaporative light scattering, infrared (IR), transport, aerosol-based, and chiral and pulsed amperometric [32].

FT-IR is an auxiliary tool for molecular identification through the transitions between vibrational states that are mainly related to vibrations centered on functional groups that, in combination, results in a specific spectrum fingerprint unique to each compound and allows it to be distinguished from other molecules present in database libraries, such as SpectraBase, NIST, Scifinder, etc. [33].

Beyond that, with the development of methodologies for analytical tools, several platforms were applied, such as HPLC–MS/MS, UPLC coupled to high-resolution mass spectrometry (UPLC-HRMS), or UHPLC–MS/MS. The MS detector is able to an unequivocal mass identification providing structural information of the analytes, and all these platforms are widely used for physicochemical characterization of the molecular structure of pure compounds [34,35].

Along with the ability of physical separation of compounds, the HPLC–MS/MS and UPLC-HRMS are employed to identify molecular structures by analysis of their fragmentation derived from mass spectrometry. Due to their robustness, these analytical techniques are extensively used for qualitative and quantitative analysis [31,32,34,35].

An overview of the concepts and applications of the different types of chromatographic techniques was recently published, including the most recent technological advances and achievements that improved its contribution to the science and technological process in several areas, such as in agricultural and food science, environmental sciences, chemical and petrochemical industries, health sciences and medicine, pharmacology and biotechnology [32].

X-ray diffraction (XRD) and NMR spectroscopies are analytical tools for the characterization of molecular structures of pure compounds at the atomic level [35,36].

Regardless of some limitations, such as time consumption and the requirement of large amounts of crystalline samples, the use of XRD has increased in the pharmaceutical area and can be used to determine structures at the atomic level, mainly for biological macromolecules [35].

Although XRD and NMR can be complementary techniques, NMR has the advantage of being automatable, allowing high throughput for large-scale sample analysis, requiring less sample, and being able to preserve it [35,36,37]. With less than a milligram of sample, in addition to the molecular structure characterization, the NMR allows to determine the content and purity level of compounds and can be applied to characterize the molecular conformation in solution. At the molecular level, data regarding the physical properties of compounds can be obtained by NMR, such as conformational exchange, solubility, phase changes, and diffusion [36]. More detailed descriptions of the NMR fundaments and application in drug discovery and development can be found in previous reports [35,36,37].

Although not applied for stereochemical information, for structural characterization of lipids and other volatile compounds, gas chromatography/mass spectrometry (GC-MS) is the most recommended analysis [38]. Although the development of pharmaceutical drugs from volatile organic compounds (VOC) is not viable, VOCs can act as autoinducers, defend against competing species and play essential roles in attracting pollinators for spreading fungal spores.

GC/MS analytical methods are very useful for application in pharmaceutical development to detect unknown molecules and impurities in the drug product.

Bacteria, Eukaryota, and Archaea can produce and respond to chemical signals called autoinducers (AIs) that are extracellular small diffusible molecules [39]. These compounds have low molecular mass (100–300 Daltons, up to C20), generally with a low boiling point and high vapor pressure [40]. Several of these small VOCs play important roles in signaling in intra- and inter-species to kingdom and domain interactions, survival, or virulence. This cell-to-cell communication through AIs is known as quorum sensing (QS) and occurs mainly in the presence of high microbial cell population density [41].

In the presence of a sufficient concentration of AIs in the extracellular medium, the activation of a cognate response regulator can occur [42]. For example, in a community of fungi and bacteria in soil, the bacterial population can be combated by fungi for nutrition, space, or pathogenicity by the action of mycotoxins and QS inhibitors (enzymes and other chemicals) [43].

Previous studies showed that fungal QS can regulate important functions such as pathogenesis, morphogenesis, and filamentation by the action of QS molecules such as lipids (oxylipins), peptides (pheromones), alcohols derived from aromatic amino acids (tyrosol, farnesol, tryptophol, and 1-phenylethanol), acetaldehydes and some VOCs [44,45,46].

The VOCs 3-octanol, 3-octanone, and 1-octen-3-ol induced conidiation in *Trichoderma* spp. in darkness culture [47]; however, at concentrations above 500 μM, the growth and conidia formation were suppressed [48].

Among 474 VOCs across 221 bacterial and fungal genera analyzed in 2020, 61 showed phylogenetic signals across bacterial domain and fungal Kingdom, 11 volatiles were phylogenetically conserved in genera from both bacteria and fungi (e.g., geosmin), whereas 17 were phylogenetically conserved in the fungal Kingdom (e.g., aristolochene) [49]. The study showed evidence that microbial VOCs can be studied by integrating metabolomic and phylogenetic data.

In general, VOCs are heterocyclic compounds, sesquiterpenoids, long-chain fatty acids, and aromatics. Among them, the compounds 1-octen-3-ol, 3-octanone, and 2-pentylfuran are considered common fungal VOCs.

The AIs molecules are not generally strain-specific, and a huge diversity of them has been reported in fungi. As an example, the acyclic sesquiterpene alcohol farnesol is involved in the filamentation of the pathogenic polymorphic yeast *Candida albicans* reducing its ability for the yeast-to-hyphal switch, being able to inhibit hyphae formation [50], as well as tryptophol and 1-phenylethanol [51].

Farnesol also affects the growth of other yeasts and fungi, including *Candida tropicalis* and *Candida parapsilosis*, *Sacharomyces cerevisiae*, *Aspergillus fumigatus*, and *Aspergillus nidulans* [52]. Moreover, in *S. cerevisiae* the morphogenesis is regulated during nitrogen starvation by the aromatic alcohols, 1-phenylethanol, and tryptophol that act as quorum sensing molecules (QSMs) [45].

Still in *C. albicans*, the aromatic alcohol tyrosol acts as a QSM controlling the growth, morphogenesis and biofilm formation [45]. Moreover, in the yeast form, *Histoplasma capsulatum* produces some specific cell wall polysaccharides α-(1,3)-glucan, required for its virulence [53].

Since different organisms employ molecular mechanisms to block the QS perception and related functions, quorum quencher (QQ) molecules are being prospected as antimicrobial compounds to target the QS and inhibit its function [54,55,56].

In the last years, more than 2000 VOCs produced by around 1000 bacterial and fungi species have been identified and systematically organized in the mVOC 2.0 database [57,58]. The database consists of VOCs collected from ca. 300 original publications and allow searching for specific microbial VOCs by name, molecular formula, molecular weight, chemical classification, Pubchem ID, bacterial, or fungal species [59]. The search using a microorganism species will indicate all the VOCs produced by that specific species.

In drug discovery, the main analytical tools have advantages and disadvantages regarding sensitivity and costs, and in general, are applied conjointly, in order to conclude a structural characterization of organic compounds [3,32,33,34,35,36,37,38,60,61].

Liquid-chromatography-high-resolution mass spectrometry (LC-HRMS) and NMR are advanced, highly sensitive, and selective analytical tools. Used in metabolomic studies, the NMR and MS techniques are able to detect samples in the range of 1 µM and 10 nM, respectively [37]. These tools offer the advantage of comparing the obtained data with wide spectral databases, such as MS-Finder, METLIN, National Institute of Science and Technology (NIST), Dictionary of Natural Products (DNP), AntiBase, DrugBank, FooDB, Chemical Entities of Biological Interest (ChEBI), and others [37,60].

As it is known, the purification of bioactive compounds involves several bioassay-guided steps and usually takes some time. To accelerate natural-product-based drug discovery, many natural product research groups are working with prefractionated natural product libraries [61]. For this purpose, solid-phase extraction (SPE) is a useful technique that has been successfully applied for metabolite fractionating [24]. This methodology is based on the separation of the crude extract into several fractions in C18 or C8 cartridges and water/methanol step gradients. The obtained fractions can be evaluated for biological activity in cell-based and/or cell-free assays [62].

In this regard, the National Cancer Institute (NCI) launched the Program for Natural Products Discovery (NPNPD) with the goal of generating prefractionated samples (up to 1,000,000) for modern high-throughput screening technologies (HTS) and developing integrated analytical resources for rapid isolation and structure elucidation of biologically active natural products [63]. The NPNPD platform provides 45 mg of a compound with a purity of ∼97% in only 2 days, while by using traditional methods, the procedure would take 2–3 weeks. The NPNPD proposal has the potential to significantly reduce natural-product-based active principle identification timelines, cost of screening, and enable faster outcomes for assaying and identifying natural products in the HTS platform [63].

In the NPNPD platform [63], natural products extracted from plants, marine invertebrates, and microbial are fractionated through a high-throughput and automated SPE system into seven fractions of decreasing polarity. The available NCI library is an important source of new drugs and drug leads that can be accessed, free of charge, by a single principal investigator of a single project or by research groups or screening centers under completing and signing of a Material Transfer Agreement (MTA). Launched in 2019, the NPNPD library already has 326,000 natural product fractions, and the project purpose is to add new fraction sets at each consecutive year until reaching 1,000,000 fractions. Detailed information on the NPNPD platform is available at the National Cancer Institute Website (https://dtp.cancer.gov/organization/npb/npnpd_prefractionated_library.htm, accessed on 25 October 2022).

## 4. Aspects of Secondary Metabolism Pathways in Microorganisms

In microorganisms, complex biosynthetic pathways depend on gene regulation, enzymes, substrates, cofactors, and intermediates to form end products [13,14].

The SMs are represented mainly by polyketides, nonribosomal peptides (NRPs), ribosomal peptides, terpenes, shikimate-derived, and compounds that emerged from hybrid pathways [13,14,64,65,66,67,68]. The classification of these molecules is related to the primary metabolites from which they are derived and built through building blocks, forming molecules that are more complex.

The cellular regulatory elements and mechanisms involved in the biosynthetic pathways of SMs by microorganisms were previously very well discussed [13,14,66,67,68]. Particularly in fungi, the SMs are chemical classes classified according to their starter substrates (acyl-CoA, amino acids, nucleotides, carbohydrates, etc.), which are commonly incorporated in the final structure by specific enzymes, such as polyketide synthases (PKSs), nonribosomal peptide synthetases (NRPSs), dimethylallyl tryptophan synthetases (DMAT), geranylgeranyl diphosphate synthetases (GGPS), prenyltransferases, and terpene cyclases (TC) [13,14,65,66,67,68].

**Figure 3:**
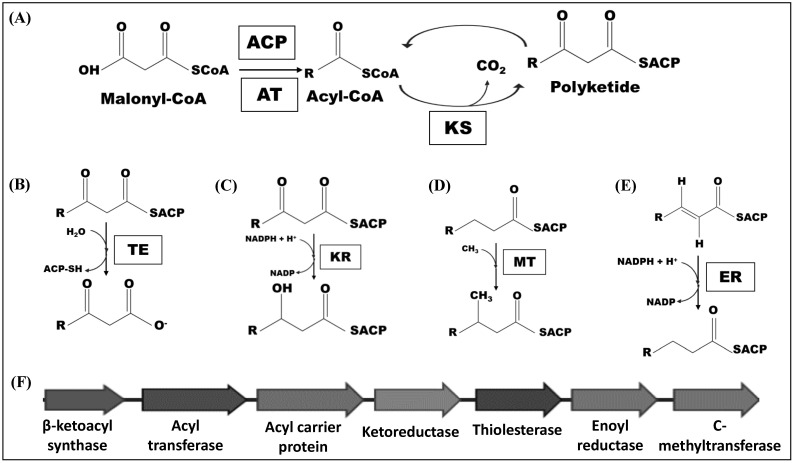
(**A**) Scheme of polyketide biosynthesis by polyketide synthetase. AT—Acyl transferase; ACP—Acyl carrier protein; KS—β-ketoacyl synthase; R—Carbon Chain. (**B**–**E**) Scheme of tailoring enzymes action on polyketide chain. TE—Thiolesterase; KR—Ketoreductase; MT—C-methiltrasferase; ER—Enoylreductase, respectively. (**F**) Genetic representation of gene cluster of polyketide synthetase and tailoring enzymes.

Polyketides are part of the most diverse class of SMs and are formed through the polymerization of acyl-coA and malonyl-coA units [69,70,71,72,73,74] by the action of the enzyme complexes called PKS, briefly represented in Figure 3. Polyketides are formed by three main β-ketoacyl synthase domains, responsible for the Claisen condensation and acyl transferase, which performs the transfer of the new building block to the nascent molecule and the acyl carrier protein domain, which acts as an anchor, binding covalently.

Modifications in the carbon skeleton of polyketides are carried out by tailoring enzymes, among which the main domains are thiolesterase, C-methyltransferase, enoyl reductase, and ketoreductase. These enzymes are responsible for the hydrolysis of thiol groups, the addition of a methyl group at the α position, and the reduction of the double bond of the enoyl-ACP group and the β-ketoacyl domains, respectively [71,72,73,74].

Fungi produce the greatest diversity of NRPs, among which are the β-lactams antibiotics, such as penicillin and cephalosporin [75,76]. The biosynthesis of these compounds is similar to that of polyketides. The NRPSs enzymatic complex also has three main domains. The adenylation domain (A), which through ATP will adenylate amino acids; the peptidyl carrier protein domain (T), responsible for the binding of a thioester group to the nascent chain; and finally, the condensation domain (C), which catalyzes the peptide bonds in the nascent chain and the amino acids [77,78]. After the beginning of the synthesis, structural changes such as epimerization, n-methylation, cyclization, acylation, glycosylation, hydroxylation, and halogenation occur [79].

An emerging class of SMs is small peptides, known as ribosomally synthesized and posttranslationally modified peptides (RiPPs) [80,81]. These compounds produced by fungi can be applied as antimicrobial and antiviral [82]. The RiPPs biosynthesis via the ribosome is differentiated from NRPS through post-translational modifications such as cyclization, hydroxylation, modifications in amino acid residues, and formation of new bonds [83,84].

Terpenoids are derived from the organic compounds obtained via the mevalonate pathway, isopentenyl diphosphate (IPP), and dimethylallyl diphosphate (DMAPP). The terpene biosynthesis pathway comprises condensation steps catalyzed by enzymatic complexes called terpene synthase, which will synthesize monoterpenes using the precursor geranyl disphophates (C10), sesquiterpenes from the precursor farnesyl diphosphate (C15), and diterpenes from geranylgeranyl diphosphate (C20). Compounds with C30 or C40 are formed by the precursors’ squalene and phytoene, respectively [85,86]. These linear chains undergo modifications via enzyme complexes, prenyl transferase, terpene cyclases, and other terpene synthases, resulting in dephosphorylations, condensation, and cyclization of the hydrocarbon backbone [78].

As mentioned above, it is well known that genes encoding the enzymes for the biosynthesis and transport of SMs and those for regulating pathway-specific are encoded in the genome, forming biosynthetic gene clusters (BGCs) [14]; however, under standard laboratory conditions, most BGCs remain silent or are expressed only weakly [87]. To awaken or activate the expression of cryptic synthetic pathways and increase the biosynthesis of SMs by fungi, some strategies such as One-Strain Many-Compounds (OSMAC), Microbial Co-culture, Chemical Epigenetic Modification, and Molecular-Based approaches are being carried out [4,14]. These strategies were previously very well discussed, and all of them have strengths and weaknesses [13,14].

Epigenetic modifications in BGCs comprehend chromatin remodeling by histone posttranslational modifications, DNA methylation, and RNA interference. Therefore, in this direction, small-molecule epigenetic modifiers are able to suppress or activate the associated enzymes, resulting in the regulation of the expression of specific genes that encode the SMs biosynthetic pathways in fungi. Among them is the DNA methyltransferase inhibitors (DNMT) 5-azacytidine, 5-aza-2′-deoxycytidine (decitabine), hydralazine hydrochloride, and the histone deacetylase (HDAC) inhibitors suberoylanilide hydroxixamic acid (vorinostat or SAHA), suberoyl bishydroxamic acid (SBHA), trichostatin A, trapoxin B, sodium butyrate, valproic acid, and nicotinamide [4]. These inhibitors are effective in low concentrations or in association. Regulation of genetic expression is an approach that relies on genome mining of the target strain to awaken cryptic BGCs using bioinformatics tools for the regulation of the SMs biosynthesis [65].

In fungi from distinct sources, BGCs play an important role in the production of chemical skeletons and structures with wide possibilities of bioactivities. Most of these gene clusters encode SMs; however, they are silent under laboratory conditions. To activate the biosynthetic potential of these genes, many methods or tools at the genome, proteome, transcriptome, or metabolome have been developed [14,65]. For this purpose, microorganism co-culture is an effective strategy in which, cell–cell contact, the chemical signal between the cells, or even biotransformation of one metabolite by another microorganism are possible mechanisms able to increase the production or activate the synthesis of novel SMs [88].

## 5. Bioactive Fungal Secondary Metabolites and Derivatives Successfully Applied by the Pharmaceutical and Agrochemical Companies

In microorganisms, the SMs are produced as a strategy to enhance their growth or even to improve their defenses or survival ability; however, SMs produced by fungi had their importance highlighted mainly after the isolation of penicillin **(1)** by Fleming, from the fungus *Penicillium chrysogenum* [89], lately re-identified as *Penicillium rubens* [90].

**Figure 4:**
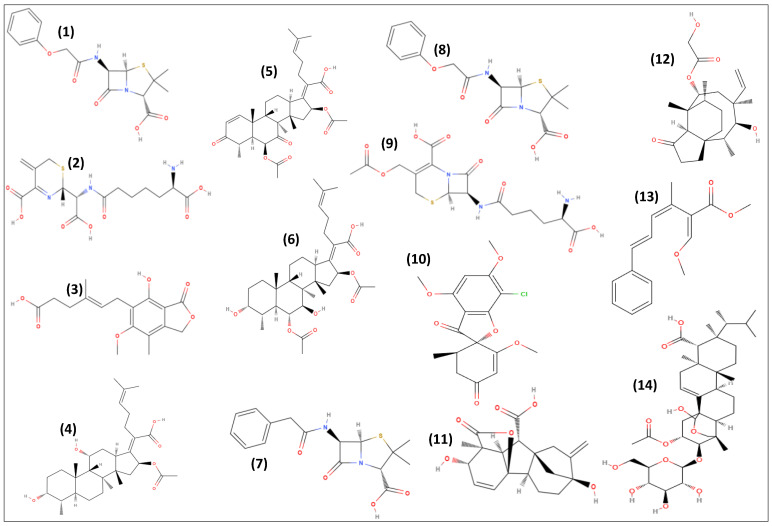
Examples of secondary metabolites from fungi and some of their derivatives successfully applied by pharmaceuticals and agrochemicals industries. **(1)** penicillin, **(2)** cephalosporin, **(3)** mycophenolic acid, **(4)** fusidic acid, **(5)** helvolic acid, **(6)** cephalosporin P1, **(7)** penicillin G, **(8)** penicillin V, **(9)** cephalosporin C, **(10)** griseofulvin, **(11)** gibberellic acid, **(12)** pleuromutilin, **(13)** strobilurins A, and **(14)** oudemansins. Structures were obtained from https://molview.org/ (accessed on 2 November 2022).

**Figure 5:**
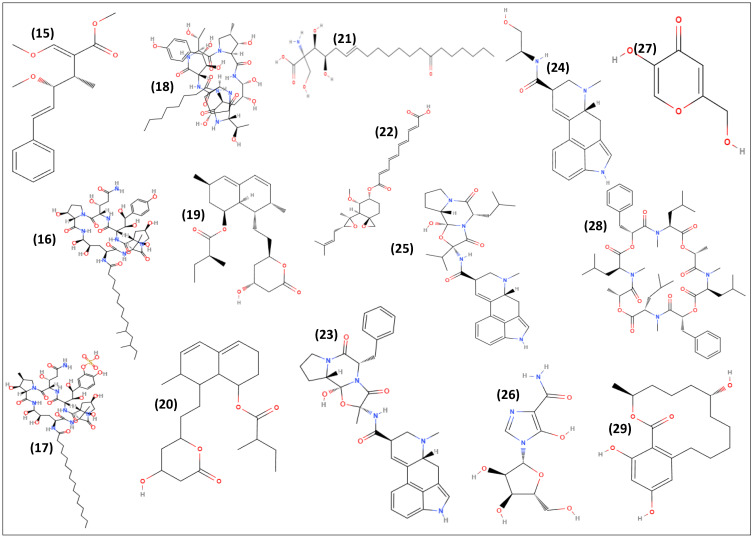
Examples of secondary metabolites from fungi and some of their derivatives successfully applied by pharmaceuticals and agrochemicals industries: **(15)** pneumocandin B0, **(16)** FR901379, **(17)** echinocandin B, **(18)** enfumafungin, **(19)** lovastatin, **(20)** compactin (ML-236B), **(21)** myriocin (ISP-I), **(22)** fumagillin, **(23)** ergotamine, **(24)** ergometrine, **(25)** ergocryptine, **(26)** mizoribine, **(27)** kojic acid, **(28)** PF1022A, and **(29)** α-Zearalanol (α-ZAL). Structures were obtained from https://molview.org/ (accessed on 2 November 2022).

The interaction of SMs with biological receptors can be favored due to the high diversity and specificity of these molecules, and Figure 4 and Figure 5 illustrate a few examples of SMs and their derivatives successfully applied by pharmaceuticals and agrochemicals industries.

In the last years, due to studies on fungal biology, the importance of fungal SMs has been more widely recognized, and their pivotal participation in the survival, defense, and adaptation against biotic and abiotic conditions is well described. Over time, several different SMs were discovered, such as statins, cephalosporin **(2)**, and mycophenolic acid **(3)**, thus expanding and encouraging the studies for the discovery of more bioactive compounds by pharmaceuticals and agrochemicals companies [5,6,7,8,9,10,15,91].

Fusidic acid **(4)** was described in 1962 and is produced by the fungus *Fusidium coccineum* [92]. Commercialized as Fucithalmic, Fucidin, Usidin, Fucicort, Fucibet, or Taksta this antibiotic is indicated for treatments of topical or eye infections caused by Gram-positive bacteria, including methicillin-resistant *Staphylococcus aureus* acting by inhibiting translocation during protein synthesis [93]. Recent studies are in progress to the discovery and synthesis of more active analogs of fusidic acid **(4)**, including compounds with anticancer activity [94]. Interestingly, fusidic acid **(4)**, helvolic acid **(5)**, and cephalosporin P1 **(6)** are fusidane-type antibiotics that share a common early stage biosynthetic route catalyzed by six conserved enzymes, which are then divergent to generate fusidic acid **(4)**, helvolic acid **(5)**, and cephalosporin P1 **(6)**, respectively, under the action of different post-modification enzymes [95].

Penicillins **(1)** and cephalosporin **(2)** are known antibacterial compounds that interfere with cell wall peptidoglycan biosynthesis and are used in the treatment of Gram-positive and some Gram-negative bacterial infections. Penicillins G **(7)** and V **(8)** are NRPs obtained from *P. rubens* and resulted in the development of benzylpenicillin and phenoxymethylpenicillin. The semisynthetic penicillins (piperacillin, amoxicillin, ampicillin) are derived from the acylation of 6-aminopenicillanic acid derived from penicillin G **(7)**. Cephalosporin C **(9)** is also an NRP produced by *Acremonium chrysogenum,* and from its structural core 7-amino cephalosporanic acid several derivatives such as cephalothin, cephalexin, cephradine, and cefadroxil were obtained [15].

A (Antibiotic S 7481F1, Ramihyphin A, CsA) is also an NRP produced by SmF of the aerobic fungus *Tolypocladium inflatum* [96], and it is available in the drug market as Sandimmune, Neoral, Restasis, Gengraf or as a generic drug. Cyclosporin A acts by binding to cyclophilin A, resulting in the inhibition of calcineurin, which activates the transcription of interleukin 2. This drug has been used as immunomodulatory to prevent organ transplantation and tissue graft rejection [97].

The fungi *Penicillium griseofulvum, Penicillium aethiopicum,* and *Penicillium coprophilum* are sources of the halogenated polyketide griseofulvin **(10)**, which is present in the commercial products Fulcine, Fulsovin, and Grisovin. This antifungal agent inhibits mitosis by binding to tubulin and preventing tubulin polymerization and has been used in the treatment of fungal infections of the skin, hair, and nails [98].

The diterpene gibberellic acid **(11)** is produced by the fermentation of the fungus *Fusarium fujikuroi*. This hormone is commonly used for plant growth through plant tissue culture and applications for certain high-value crops [99].

Another interesting SM is the diterpene pleuromutilin **(12)**, produced by the fungi *Clitopilus passeckerianus* and *Clitopilus* spp. This molecule is a precursor of the partial chemical synthesis of the topic antibiotic retapamulin (Altabax) and of tiamulin and valnemulin (Econor). The two latter compounds are for animal use and have been used for topical treatment of Gram-positive bacterial infections in cattle, acting by targeting the peptyl drug transferase center of the bacterial ribosome [100].

Strobilurins A-D **(A = 13)** and oudemansins **(14)** are polyketides structurally related to an initial unit of benzoyl CoA and are produced by *Strobilurus tenacellus*. Applying Quantitative Structural Activity Relationship (QSAR) on the structures of the natural strobilurins, many pesticide companies discovered several synthetic analogs that are more efficacious and stable fungicides, including the synthesis of fenamidone, azoxystrobin, kresoxim methyl, fluoxastrobin, trifloxystrobin, pyraclostrobin—carbamate, picoxystrobin, and dimoxystrobin [101]. Part of these compounds is registered worldwide for agrochemical use, and others are in the process of registration. This class of fungicides, for phytopathogenic fungi acts on the mitochondrial cytochrome b, which interferes with the respiratory electron transport in fungi.

Pneumocandin B0 **(15)** is an NRP acylated to polyketide and produced by *Glarea lozoyensis*, being the precursor of the partial chemical synthesis of the antifungal echinocandin, caspofungin (MK-0991) [102].

The fungi *Coleophoma cylindrospora* or *Coleophoma empetri* F-11899 produces the NRP acylated to fatty acid, coded as FR901379 **(16)**. This sulfated echinocandin is a precursor of the partial chemical synthesis of the echinocandin, micafungin, commercially sold as Mycamine and Fungiguard [103]. Recently, the BGCs involved in the biosynthesis of FR901379 **(16)** were analyzed and an efficient clustered regularly interspaced short palindromic repeats/Cas9-based gene editing tool for the industrial production strain *C. empetri* SIPI1284 was established [104].

The fungi *Aspergillus pachycristatus* and *A. nidulans* from the *Nidulants* section produce a molecule identified as echinocandin B **(17)**. This compound is classified as a fatty acid-acylated NRP, which is a semi-synthetic echinocandin synthesized from the fermentation product of *Aspergillus* sp. anidulifungin, which has been marketed as Eraxis [105].

The triterpene glycoside, enfumafugin was isolated from *Hormonema carpetanum*, an endophytic isolated from leaves of *Juniperus communis* and showed high antifungal activity *in vitro* against *Candida* sp. and *A. fumigatus*, and moderate efficacy in an *in vivo* mouse model of disseminated candidiasis [106]. Modifications in the structure of this compound to improve the oral bioavailability and pharmacokinetic properties led to the development of a semi-synthetic derivative named ibrexafungerp (IBX, MK-3118, SCY-078), which is currently in phase III clinical trials [107].

As well as caspofungin, micafungin, and echinocandin B **(17)**, enfumafungin **(18)** inhibits 1,3-beta-D-glucan synthesis, a major structural component of the fungal cell wall, which is not present in mammalian cells, consequently avoiding toxicity problems, and is used in the treatment of systemic fungal infections.

Lovastatin **(19)** (mevinolin, monacolin K, L-154803, MK-803, ML-530B) is a bioactive polyketide commercially sold as Mevacor. Its semisynthetic product, simvastatin, is on the market as Zocor and Simvador. This statin blocks 3-hydroxy-3-methylglutaryl-coenzyme A reductase, and prevents the conversion of mevalonate into cholesterol, being the most frequently used drug in the treatment of hypercholesterolemia to reduce the risk of cardiovascular diseases and to manage abnormal lipid levels by inhibiting the endogenous production of cholesterol in the liver [108]. Additional studies have indicated several important applications for lovastatin **(20)** as an antimicrobial and agent for treatments of cancers and bone diseases [19,108].

Lovastatin **(19)** biosynthesis has been reported in several fungal species, including *Aspergillus terreus*, *Monascus purpureus*, *Penicillium citrinum*, *Paecilomyces viridis*, *Penicillium purpurogenum*, *Pleurotus* sp., and *Trichoderma viride*; however, *A. terreus* is the species used for industrial production. Furthermore, to elucidate the lovastatin **(19)** biosynthesis, most genomic, and transcriptomic studies were performed in *A. terreus* ATCC 20542. Studies regarding the biosynthetic pathway showed that lovastatin **(19)** is synthesized from two-chain reactions using acetyl and malonyl-CoA and methionine as substrates through the biosynthetic pathway PKS. Two key multifunctional enzymes are involved in biosynthesis, which are encoded by specific genes organized in clusters on the fungal genome [19,108].

In the group of statins, compactin (ML-236B) **(20)** produced by the fungi *P. citrinum* and *Penicillum solitum* also reduces the risk of cardiovascular diseases and is used as a substrate for the biotransformation or partial chemical synthesis of mevastatin. Commercially, it is available as Pravachol, Selektine, or Lipostat. This statin inhibits 3-hydroxy-3-methylglutaryl-coenzyme A reductase and is widely used for the treatment of hypercholesterolemia by preventing the conversion of mevalonate into cholesterol [109].

Myriocin (ISP-I) **(21)** is an amino acid lipid produced by the fungus *Isaria sinclairii*, which structural model is used as a template for the synthesis of fingolimod, commercially available as Gilenya. Used in the treatment of multiple sclerosis through the activation of its structure, this drug connects with extracellular G protein-coupled receptors, preventing the release of lymphocytes that enter the central nervous system [110].

Mycophenolic acid **(3)** is an immunosuppressive drug used to prevent organ transplant rejection. Synthesized by the fungus *Penicillium brevicompactum*, this compound, classified as a meroterpenoid, is used to produce mycophenolate mofetil and mycophenolate sodium, which are commercially available as Cellcept and Myfortic. This drug blocks inosine monophosphate dehydrogenase (IMPDH), consequently causing a decrease in DNA synthesis [91].

Another meroterpenoid is the compound fumagillin **(22),** which is produced by the fungus *A. fumigatus* and is commercially available as dicyclohexyl ammonium fumagillin **(22)** (Fumidil B^®^). This compound is a methionine aminopeptidase2 (MetAP2) inhibitor used to control Nosema disease in bees caused by *Nosema apis* [111].

The alkaloids ergotamine **(23)**, ergometrine **(24)**, and ergocryptine **(25)** are prenylated NRPs obtained as SMs from the fungi *Claviceps purpurea, Claviceps fusiformis,* and *Claviceps paspali*.

Ergotamine **(23)** acts as a vasoconstrictor, being used in migraine attacks, and is commercially available as ergotamine **(23)** tartrate (Ergomar and Migril), dihydroergotamine mesylate (Migranal), dihydroergotamine (DHE 45), or associated with caffeine (Cafergot, Cafetrate). This alpha-1 selective adrenergic agonist vasoconstrictor is an agonist of 5-OH tryptamine (5-HT2) receptor 1B, norepinephrine, dopamine, and serotonin receptors. This ergot alkaloid is used as an anti-migraine agent, also associated with belladonna and phenobarbital, for the relief of menopausal hot flashes [112].

The synthetic derivative ergometrine **(24)** maleate is commercially available as Ergotrate and Ergovin, and the derivative methylergometrine can be combined with oxytocin (Syntomethrin) for veterinary use. Both ergometrine **(24)** and methylergometrine are used for the treatment of postpartum hemorrhage. Ergometrine **(24)** is a serotonin antagonist, activating or blocking 5-HT2 receptors and used as the precursor for partial chemical synthesis of lysergic acid diethylamide, LSD-25, and LSD (Delysid) [113].

The chemical structure of ergocriptine **(25)** is the core for the partial chemical synthesis of bromocriptine (Parlodel), an agonist of dopamine D2 and various serotonin and adrenergic receptors used in the treatment of reproductive disorders (galactorrhea, prolactin-dependent mammary carcinoma, amenorrhea, acromegaly, or anovulation) or complications such as osteoporosis. Bromocriptine is also an option for the treatment of Parkinson’s disease [114].

The imidazole nucleoside mizoribine **(26)** is produced by the ascomycete *Penicillium brefeldianum* [115] and is the active component of Bredinin™ OD25 and Bredinin™ OD50, marketed since 2016 by the company Asahi Kasei Pharma as an immunosuppressant for renal disorders and transplant.

The fungi *Aspergillus oryzae*, *Aspergillus tamarii*, and *Aspergillus flavus* are producers of the kojic acid **(27)**, classified as a pyrone, derived from glucose. In industry, it is often formulated as kojic dipalmitate to increase stability and shelf life. As an inhibitor of tyrosinase, the kojic acid **(28)** suppresses melanogenesis and is used as an antioxidant in cosmetic products to lighten skin color and treat abnormal hyperpigmentation [116].

PF1022A **(28)** is an NRP produced by the fungus *Rosellinia* sp. and its derivative, bisparamorphonyl, is produced commercially as Emodepside. This form disrupts the function of the target neuromuscular nematode by the interactions with the latrophilin, such as HC110-R G protein-coupled and SLO-1 potassium channels. This NRP can be associated with the anthelmintic drug praziquantel to treat roundworms, hookworms, and tapeworms in cats [117].

α-Zearalanol **(29)** (α-ZAL) is a polyketide produced by *Fusarium* spp., including *Fusarium culmorum*, *Fusarium equiseti*, *Fusarium sporotrichioides*, and *Fusarium semitectum*. This estrogenic mycotoxin is obtained from the fermentation of zearalenone by a highly reducing and non-reducing PKS, followed by hydrogenation to form α-zearalanol **(29)**. The synthetic version of α-ZAL, zeranol, is marketed as a growth promotor in the United States and Canadian livestock industries, whereas experimental animal models have typically demonstrated impaired growth following prenatal exposure [118].

In addition to the fungal SMs with proven biological effects, there are several others in the study by different groups in many countries for application as cytotoxic or antimicrobial agents, and a few of them are discussed in the next topics.

## 6. Studies of Fungal Secondary Metabolites as Cytotoxic and Antimicrobial Agents

### 6.1. Cytotoxic Compounds

Cancer has been the leading cause of death and an important barrier to increasing life expectancy around the world [119]. According to the World Health Organization [120], in 2018, cancer was the second leading cause of death globally. It was responsible for at least one in every six deaths, accounting for at least 9.6 million deaths. An estimate for 2020 indicates that there were 19.3 million new cases of cancer and 10.0 million deaths worldwide [121]. In Brazil, it is estimated that between 2020 and 2022, there will be around 625,000 new cases of cancer, the most frequent being non-melanoma skin cancer, followed by breast, prostate, colon, rectum, lung, and stomach cancer [122].

Despite the progress in research for cancer treatments, which has improved the life expectancy and cure of many patients, the complications from the adverse effects due to chemotherapy and radiotherapy are disastrous. In addition, there are cases of chemoresistance and cancer recurrences.

Currently, there is a great demand for new oncological therapies that reduce or do not cause severe adverse effects to patients, providing an improvement in quality of life. In this context, several studies seek new antitumor molecules from natural sources, attempting to reduce treatment costs, increase specificity, and decrease the side effects [123].

Among all anticancer drugs described from 1946 to 2019, in a total of 321, about 71% (230) are based on or derived from natural products [1]. According to Keller [65], from 1993 to 2001, among the 1500 fungal SMs, 50% were described as antimicrobial and antitumor compounds.

Various tumor cell lines from human and animal sources have been useful and applied for *in vitro* cytotoxicity and *in vivo* studies of the anticancer potential of natural products, whether by the academy or by pharmaceutical companies.

The main human tumor cells applied are from lung carcinoma (A549, HL251, 95-D, NCI-H460), melanoma (A375-S2, SK-MEL-28), glioblastoma (SF268, U-251, U373), caucasian esophageal squamous carcinoma (OE21), oligodendroglioma (Hs683), colorectal carcinoma (HCT 116, HT29), prostate adenocarcinoma (PC-3, PC-3M), leukemia (HLK 210, Jurkat), acute promyelocytic leukemia (HL-60), chronic myelogenous leukemia—CML (K562), breast carcinoma or adenocarcinoma (BT-20, BT-474, MDA-MB-435, MDA-MB-468, MCF7, MCF7-dox T47D, MDA-MB-231, SK BR-3), cervical carcinoma (HeLa), hepatocellular carcinoma (HepG2, MHCC97H, BEL-7402), gastric carcinoma (MGC-803), ovarian carcinoma (HO-8910), and neuroblastoma (SH-SY5Y). Among mouse cells are melanoma (B16-F10), lymphoma (L5178Y), and lymphocytic leukemia (L1210) [5,124]. Many other specific cell lines are used or were mentioned throughout this review discussion.

Several fungal SMs are reported as revealing potential anticancer activity through different mechanisms of action, such as apoptosis, angiogenesis, cell cycle regulation, metastasis, and signal transduction cascades [124]. Table 1 presents several examples of cytotoxic fungal SMs already described.

In the last years, various fungal bioactive SMs have been described [5,6,7,8,9,10,125], such as paclitaxel. Paclitaxel was initially discovered from the medicinal plant *Taxus brevifolia*, and its cytotoxicity and apoptotic effect were studied *in vitro* on different cancer cell lines such as BT220 (breast), H116 (colon), Int407 (intestine), HL251 (lung), and HLK210 (leukemia) and MCF-7 (breast cancer). Paclitaxel, commercially called Taxol^®^, was approved by the United States Food and Drug Administration (FDA) Agency in 1992, and is clinically applied in the treatment of breast, throat, neck, lung, and ovary cancer [126]. To attend to the demand, paclitaxel is produced by a fermentation process of *Taxus* sp. leaves in aqueous media, and despite the huge progress in the biotechnological approaches, *Taxus* spp. cell culture productivity remains a big challenge [127].

**Table 1:** Examples of cytotoxic secondary metabolites produced by fungi species.

Metabolites	Fungi Species	References
Bislongiquinolide (trichotetronine), dihydrotrichodimerol	*T. citrinoviride*	[124]
Paclitaxel	*B. robillardoides* *L. theobromae* *Paraconiothyrium* sp. *P. microspore* *P. neglecta* *P. versicolor* *T. andreanae*	[128,129,130,131,132,133]
Camptothecin, podophyllotoxin, vinblastine, vincristine	*T. radicus*	[134]
Altenuene, stemphyperylenol	*Botryosphaeria* *dothidea*	[135]
Alterperylenol, altertoxin II	*Alternaria* sp. A744	[136]
Alterperylenol, stemphyperylenol	*Alternaria* sp.	[137]
(-)-(10E,15S)-10(11)–dehydrocurvularin, (-)-(10E,15S)-6-Chloro-10(11)-dehydrocurvularin	*Alternaria* sp. AST0039	[138]
Phomoxanthone A	*P. longicolla*	[139]
Embellicines A and B	*E. eureka*	[140]
Pyrrocidin A	*N. ramulariae* Wollenw KS-246	[141]
Aphidicolin	*N. sphaerica*	[142]
Steroid (22E,24R)-8,14-epoxyergosta-4,22-diene-3,6-dione	*P. immersa*	[142]
12′-Hydroxyroridin E, 13′,14′-hydroxymytoxin B, 14′- hydroxymytoxin B, vertisporin, mytoxin C, roridin E, 2’,3’-epoxymyrotecin A, myrothecin A, myrotoxin A	*M. roridum*	[143]
Daldinone I	*Annulohypoxylon* sp.	[144]
Cladosporol A	*C. cladosporioides*	[145]
Engyodontiumones H, AGI-B4	*E. album* DFFSCS021	[145]
Rosoloactone	*T. roseum*	[146]
Penipacide A and E	*P. paneum*	[147]
Beauvericin	*Fusarium oxysporum* EPH2R_AA_	[148]
Bikaverin	*F. oxysporum* CEC1S	[148]
Beauvericin	*F. oxysporum*	[149]
Penichryfurans A and B	*P. chrysogenum*	[150]
Brefeldin A (BFA)	*P. brefeldianum*	[151]
Porritoxin	*A. porri*	[152]
Amides (AI-77-B, AI-77-F, Sg17-1-4)	*A. tenuis* sg17-1	[153]
Dehydroaltenusin	*A. tenuis*	[154]
Alternariol, alternariol 5-*O*-sulfate, alternariol 5-*O*-methyl ether, altenusin, desmethykaltenusin	*Alternaria* sp.	[154]
Alternol	*A. alternata var. monosporus*	[155]
Cajanol (5-hydroxy-3-(4-hydroxy-2-methoxyphenyl)-7-methoxychroman-4-one)	*H. lixii*	[156]
Sorbicillinoids and bisorbicillinoids	*P. chrysogenum* *Penicillium* sp. *P. terrestre* *Phialocephala* sp. *S. album* *Trichoderma* sp. *Trichothecium* sp.	[157,158,159,160,161,162,163,164,165,166,167,168]
Sorbicillinoids: tricoreeseione A and B, tricodermolide B, 13-hydroxytricodermolide, 24-hydroxy-tricodimerol, 15-hydroxybisvertinol Analogs of sorbicillinoids: trichodimerol, 24-hydroxy-bisvertinol, bisvertinol	*T. reesei* HN-2016-018	[169]
Deacetylcytophasin C, zygosporin D	*C. taii*	[170]
Ylarone A, (-)5-methylmelein	*X. psidii*	[171]
Diapolic acid AB, xylarolide, fomolide	*D. terebinthifolli*	[172]
Pestalrone B	*P. karstenii*	[173]
Siccayne [2-(3-methyl-3-buten-1-ynyl) hydro]	*P. fici*	[173]
Chaetoglobosins A, B, D, E, F, Fex, 20-dihydrochaetoglobosin A	*C. globosum*	[174,175]
Pensulfonoxy, Pensulfonamide	*P. aculeatum*	[176]
Polonidine A, fructigenine A	*P. polonicum* TY12	[177]
Giluterrin	*A. terreus* P63	[178]
Arvoredol	*Penicillium* sp. F37	[179]
Anhydrofusarubin, fusarubin	*Cladosporium* sp.	[180]
Xanthocillins X and Y1	*P. chrysogenum* CCTCC M 2020019	[181]

Over time, in an attempt to find a good source of paclitaxel, fungi species have been studied for this purpose. First, paclitaxel was identified as an SM of the endophytic fungus *Taxomyces andreanae*, isolated from the plant with the same name [128]. Later, it was also identified as an SM of at least 35 endophytic fungi [12].

Endophytes isolated from *Taxus wallachiana* in the Himalayas can produce paclitaxel and other chemicals with valuable antitumor activity, and among them, the fungi *Pestalotiopsis microspora* [129]. The endophytes *Bartalinia robillardoides* isolated from the medicinal plant *Aegle marmelos* [130] and *Lasiodiplodia theobromae* from *Morinda citrifolia* [131] are other examples of paclitaxel biosynthesis.

Among the fungi species able to produce paclitaxel, *Pestalotiopsis neglecta* and *Pestalotiopsis versicolor,* which were isolated as endophytic from the Japanese Yew tree, *Taxus cuspidata,* produced paclitaxel with a yield of 375 and 478 μg/L, respectively. This yield is 9560 times higher than what was previously reported as obtained for the fungus *T. andreanae* [132].

The co-culture of *Paraconiothyrium* sp., a paclitaxel-producing fungus with *Alternaria* sp., increased the paclitaxel production by 3-fold, and when the *Phomopsis* sp. was added to the coculture, the yield increased to around 7.8-fold [133].

The compounds camptothecin, vincristine, vinblastine, and podophyllotoxin, produced by plants, and their semisynthetic derivatives, are clinically used for cancer treatments (e.g., ovarian, breast, prostate, lung cancers, and leukemias). However, these compounds were also described as fungal SMs of *Talaromyces radicus* isolated from *Catharanthus roseus*, in India, with good yields of vincristine (670 μg/L) in modified M2 medium and of vinblastine (70 μg/L) in potato dextrose broth medium [136]. Similarly, the endophytic fungus *F. oxysporum*, isolated from the leaves of the same plant, also in India, produced vincristine and vinblastine, resulting in a yield of 67 and 76 mg mg/L, respectively [182].

Stemphyperylenol and altenuene were isolated from the endophytic *B. dothidea* of *Melia azedarach* and exhibited cytotoxicity against HCT116 cells with an IC50 value of 3.13 μM, while the positive control etoposide showed IC50 of 2.13 μM [135].

The compounds alterperylenol and altertoxin II were described as SMs of the endophytic fungus *Alternaria* sp. A744 derived from *Morinda officinalis*, and both compounds significantly inhibited the proliferation of the tumor cell lines MCF-7, HepG-2, NCI-H460, and SF-268, with IC50 values in the range of 1.91–9.67 µM [136]. Later, Zhao et al. [137] also reported the isolation of the two perylenequinones, stemphyperylenol, and alterperylenol, from *Alternaria* sp. endophyte from marine plants in China.

The *Alternaria* sp. AST0039, derived from *Astragalus lentiginosus* collected from a roadside area in central Arizona, in the USA, provided the resorcylic acid lactones curvularins, (-)-(10E,15S)-6-chloro-10(11)-dehydrocurvularin, and (-)-(10E,15S)-10(11)–dehydrocurvularin, and both compounds exhibited cytotoxicity to the tumor cells NCI-H460 (human non-small cell lung cancer), SF-268 (human CNS glioma), MCF-7 (human breast cancer), and PC-3M (metastatic human prostate cancer adenocarcinoma), with IC50 lower than 5.0 µM. Additionally, (−)-(10E,15S)-6-chloro-10(11)-dehydrocurvularin inhibited the growth of MDA-MB-231 (human metastatic breast adenocarcinoma) cells with IC50 of 2.95 µM) [138].

Phomoxanthone A is an SM produced by the endophytic fungus *Phomopsis longicolla* isolated from the mangrove plant *Sonneratia caseolaris* (*Lythraceae*) collected in southern China [139]. This tetrahydroxanthone atropisomer derived was cytotoxic with IC50 of 0.7, 0.8, and 5.2 µM on human esophagus Kyse510, human tongue Cal27, and human ovarian carcinoma A2780 cell lines sensitive to cisplatin, and IC50 of 0.9, 0.8 and 0.9 5.6 µM on these same cell lines, however cisplatin-resistant, respectively. Additional data showed that this compound was selective to tumor cells, showing IC50 of 61.2 μM on healthy human peripheral blood mononuclear cells (PBMCs) and of 0.1 μM on the human Burkitt’s lymphoma cell line DG75 and human T cell lymphoma cell line Jurkat [139]. Besides the pro-apoptotic activity, phomoxanthone A activated murine T lymphocytes, NK cells, and macrophages, suggesting an activation of the immune system. The association of this dual effect to combat the cancer cells could help in fighting resistance during chemotherapy.

The fungus *Embellisia eureka*, endophytic from the plant *Cladanthus arabicus*, collected in Morocco, produced the SMs pyrrolidinone alkaloids, embellicines A and B, and the bioactivity screening of these congeners indicated pronounced cytotoxicity against human chronic myeloid leukemia cells (K562) with IC50 lower than 10 µM. Embelicin B was the most cytotoxic, with IC50 of 0.92, 0.32, 0.25, and 0.21 µM after 8, 24, 48, and 72 h of treatment, respectively [140].

Uesugi et al. [141] performed a study with pyrrocidin A, a close derivative of embellicines A and B, which was isolated from the fungus *Neonectria ramulariae* Wollenw KS-246, derived from a dead twig (plant species not reported) collected in Japan. Pyrrocidin A, originally described as a natural product with antibiotic activity, exhibited cytotoxic properties against human acute promyelocytic leukemia HL60 cells with IC50 of 0.12 µM.

The diterpene aphidicolin and the steroid (22E,24R)-8,14-epoxyergosta-4,22-diene-3,6-dione were described as SMs from the endophytes *Nigrospora sphaerica* and *Papulaspora immersa*, respectively, isolated from the plant *Smallanthus sonchifolius*. These compounds were also cytotoxic to HL60 cells with IC50 of 0.27 and 1.65 µM, respectively [142].

The fungus *Myrothecium roridum*, isolated from roots of *Ajuga decumbens* collected in Fuzhou, Fujian, China, provided nine trichothecenes derivatives, which were evaluated for cytotoxicity on the human hepatocellular carcinoma cells A549, MCF-7, HepG2, and SMMC-7721 [143]. The compounds 2’,3’-epoxymyrotecin A, myrothecin A, and 12′-hydroxyroridin E were the less cytotoxic, with IC50 values in the range of 1.25–95 µM on these cell lines; however, the other six derivatives (13′,14′-hydroxymytoxin B, myrotoxin A, 14′- hydroxymytoxin B, vertisporin, mytoxin C, roridin E) were more effective, with IC50 from 1.42 to 75 nM. It is known that the 12,13-epoxy ring is the structural feature relevant to the remarkable differences in toxicity, which explains the much lower cytotoxicity of the trichothecenes 2’,3’-epoxymyrotecin A and myrothecin A.

Among the nine SMs (daldinone H, daldinone I, daldinone J, daldinone C, hypoxylonol C, daldinone B, 3,4-dihydro-3,4,6,8-trihydroxy-l(2H)-naphthalenone, (R)-scytalone, and 1-hydroxy-8-methoxynaphthalene) of the endophyte *Annulohypoxylon* sp. isolated from the mangrove plant *Rhizophora racemosa* collected in Cameroon, only daldinone I inhibited the growth of human leukemia and lymphoma cell lines, Jurkat J16 and Burkitt’s lymphoma (Ramos) with IC50 of 14.1 and 6.6 mM, respectively [144]. The cytotoxicity mechanism was described as being by induction of intrinsic apoptosis and by blocking autophagy, a potential pro-survival pathway for cancer cells.

Chromatographic processing of the crude extract obtained from the culture of the endophytic fungus *Cladosporium cladosporioides* derived from *Datura innoxia* (Jammu, India) provided the tetralone derivative, cladosporol A [145]. The cytotoxicity assay against the NCI60 panel of human cancer cells revealed its antiproliferative effect on various cancer cell lines with IC50 values from 8.7 to 15.6 µM, being more effective against the MCF-7 cell line of breast cancer [145]. The data showed that cladosporol A triggered apoptotic as well as autophagic death of human breast cancer (MCF-7) cells.

Natural diterpenoids have been reported to induce tumor cell apoptosis. In this class, the compound rosoloactone was isolated from the endophytic fungus *Trichothecium roseum*, derived from *Ginkgo biloba L*. in Shandong Province, China. Rosoloactone displayed significant cytotoxicity *in vitro*, inducing anti-proliferative and pro-apoptotic effects in human cervical cancer HeLa cells, which were associated with endoplasmic reticulum stress (ERS) and mitochondrial damage [146]. An accumulation of misfolded or unfolded proteins in the ER lumen was observed, causing excessive ERS, as well as mitochondrial damage followed by the release of cytochrome c into the cytosol, activation of caspase-3 and -9, with subsequent activation of mitochondria-mediated apoptosis. There was a simultaneous and marked ROS production [146].

*Engyodontium album* DFFSCS021, isolated from a marine sediment sample collected in the South China Sea, produces 19 polyketides, including engyodontiumones A–J, 2-methoxyl cordyol C, sydowinin A-B, pinselin, aspergillusone B, AGI-B4, diorcinol, cordyol C, and hydroxysydonic acid, which were screened for cytotoxicity, antibacterial, and antilarval activities. Among them, engyodontiumones H and AGI-B4 were cytotoxic on the human histiocytic lymphoma U937 cell line, with IC50 values of 4.9 and 8.8 μM, respectively [183].

The anthranilic acid derivatives penipacide A and E produced by *Penicillium paneum*, also isolated from marine sediment in the South China Sea, exhibited cytotoxicity on the RKO cell line (colon carcinoma), with IC50 values of 8.4 and 9.7 µM, respectively [147].

The pharmacological plants from the Brazilian biodiversity *Ageratum myriadenia*, *Palicourea tetraphylla*, *Piptadenia adiantoides*, and *Trixis vauthieri* were used as a source of 121 fungal endophytes, which were identified as belonging to the genera Alternaria, Arthrinium, Cochliobolus, Colletotrichum, Penicillium, Fusarium, and Gibberella. The fungi strains were applied for fermentation, and crude organic extracts obtaining using ethyl acetate [184]. Among the extracts, 17 (14%) were cytotoxic for at least one human cancer cell line, with IC50 values ranging from >0.2 to 25 µg/mL, and selected for future compound isolation. Later, Alves et al. [185] demonstrated that the organic extract (EtOAc/MeOH 1:1 solvent) obtained using the culture of the endophytic fungus *Dichotomophthora* sp. was cytotoxic on cell strains of colon adenocarcinoma (HCT-166) and prostate cancer (PC3), inhibiting, respectively, 100% and 96.41% of the cellular growth.

Beauvericin and bikaverin produced by the strains of *F. oxysporum* EPH2R_AA_ and CEC1S endophytes from the plants collected from the Sonoran desert *Ephedra fasciculate* and *Cylindropuntia echinocarpus*, respectively, were active showing selective toxicity toward NCI-H460 and MIA Pa Ca-2, respectively [148].

Similarly, a strain of *F. oxysporum* isolated from the bark of *Cinnamomum kanehirae* from Jiaoban Mountain, Taiwan Province, along with other SMs provided the cyclodepsipeptide beauvericin that showed cytotoxicity against PC-3, PANC-1, and A549, with IC50 ranging from 10.4 to 49.5 1.6μM [149].

Among Penicillium species able to produce cytotoxic SMs is the endophytic fungus *P. chrysogenum*, from the marine medical red alga *Grateloupia turuturu* [150]. Through *in vitro* assay, it was possible to demonstrate the cytotoxicity activity of the N-acetyl--glucosamine derivatives, penichryfurans A and B against A549, HeLa, and HepG2 cell lines. Penichryfurans A was the most effective on the HepG2 cell line with an IC50 of 9 µM.

In addition to mizoribine **(27)** (Bredinin™ OD25 and Bredinin™ OD50) and other SMs, *P. brefeldianum* produces brefeldin A (BFA), a lactone with antifungal, antiviral, and antitumor activity. BFA is able to trigger apoptosis in tumor cells, and besides its biological properties, its structural features have aroused the interest of medicinal chemistry in developing numerous analogs to improve its bioavailability and its antiproliferative effect [151].

Lou et al. [186] reviewed metabolites from *Alternaria* sp. and their bioactivities, showing several SMs with cytotoxic effects on Hela, KB, MDA-MB-435 breast cancer, and L5178Y mouse lymphoma cells. Among them, *Alternaria alternata* JS0515 was described as an endophyte from *Vitex rotundifolia* rhizomes, and in the chemical composition of its metabolism products were found phenolics, pyranones, quinones, steroids, terpenoids, and nitrogen-containing compounds, some of which showed phytotoxic, cytotoxic, antifungal, and antimicrobial activities [186,187,188,189].

The SM porritoxin from *Alternaria porri* showed antitumor-promoting activity [152], and amides (AI-77-B, AI-77-F, Sg17-1-4) from the marine fungus *Alternaria tenuis* sg17-1 were cytotoxic on human malignant A375-S2 and human cervical cancer Hela cells in the range from 0.02 to 0.4 mM [153]. Alternariol and its derivatives from *Alternaria* sp., including alternariol 5-*O*-sulfate, alternariol 5-*O*-methyl ether, altenusin, and desmethykaltenusin were described as cytotoxic on mouse lymphoma cells (L5178Y), with IC50 of 1.7 to 7.8 µg/mL [154]. Alternariol was described as topoisomerase I and II poison, which can interfere with the impairment of DNA integrity in human colon carcinoma cells [190]. Alternariol and its 9-methyl ether induced cytochrome P450 1A1 and apoptosis in murine hepatoma cells dependent on the aryl hydrocarbon receptor [191]. Dehydroaltenusin obtained from *A. tenuis* showed specific inhibition of eukaryotic DNA polymerase α to show its high cytotoxicity on tumor cells [192].

Liu et al. [155] demonstrated for the first time that alternol, a compound purified from the metabolism of the fungus *A. alternata var. monosporus*, from yew bark (*T. brevifolia*), inhibits proliferation and induces apoptosis in mouse lymphocytic leukemia L1210 cells. Alternol induced activation of caspase-3 and caspase-9, but not caspase-8. In addition, there was a significant increase in ROS production, which may also play a role in apoptosis. Based on this study, *A. alternata* was suggested as an interesting source for its ability to produce the cytotoxic compound alternol.

A study carried out by Fernandes et al. [193] demonstrated the cytotoxic potential of SMs from the endophytic fungus *A. alternata* isolated from coffee leaves of *Coffea arabica L*. Through *in vitro* assay, the organic crude extract obtained using dichloromethane showed cytotoxicity with IC50 of 400 µg/mL on human cervical cancer cells (HeLa).

In another study, Bhimba et al. [194] showed that the organic extract obtained from the culture of the fungus *Hypocrea lixii*, isolated from leaves of the mangrove *Rhizophora mucronata*, *Avicennia officinalis*, and *Avicenna marina*, displayed cytotoxic activity on human hepatocellular carcinoma (HepG2) and breast cancer (MCF-7) cells. This extract also presented antimicrobial effects on *Bacillus subtilis*, *S. aureus*, *Escherichia coli*, *Proteus vulgaris*, and mainly on *Pseudomonas aeruginosa*. Following that, a study published by Zhao et al. [156] demonstrated that the endophytic fungus *H. lixii*, isolated from the medicinal plant pigeon pea (*Cajanus cajan* [L.] Millsp.), in China, produces the SM cajanol, which showed cytotoxicity on human lung carcinoma cells (A549).

Cajanol, 5-hydroxy-3-(4-hydroxy-2-methoxyphenyl)-7-methoxychroman-4-one, is an isoflavone, and previous studies showed its antiplasmodial activity [195] and inhibition of prostate-specific antigen secretion in LNCaP cells [196]. Despite its pharmacological potential, cajanol occurrence is rare. Among 135 endophytic fungi isolated from roots, stems, and leaves of pigeon pea, only three strains of *H. lixii* were able to produce cajanol [156].

Sorbicillinoids (also called vertinoids) are hexaketide metabolites, first isolated in 1948 as an impurity in penicillin **(1)** [197]. This family of compounds has more than 100 analogs already described, having a specific carbon skeleton of sorbicillin, in which the cyclization has taken place on the carboxylate terminus [198,199,200]. According to their basic structural features, sorbicillinoids are classified as monomeric, dimeric, trimeric, polycyclic, and vertinolides [198,199,200]. Due to their unique structures, sorbicillinoids have received particular attention for drug development in the pharmaceutical and agrochemical areas.

The structures of 91 sorbicillinoids were reviewed, including those from 62 new sorbicillinoids obtained from fungi between 2016 and 2021, as well as their bioactivities as anti-inflammatory, antiviral, radical scavenging, cytotoxicity, antimicrobial, anti-inflammatory, phytotoxic, and α-glucosidase inhibitory [198].

These classes of SMs have been isolated from terrestrial or marine fungi. Among the sorbicillinoids producers, the species *Aspergillus* sp., *P. citrinum*, *Penicillium notatum*, *Emericella* sp., and *Phaeoacremonium* sp. belong to the Trichocomaceae Family. In the class of Sordariomycetes, the sorbicillinoid producers are *Trichoderma* sp., *T. viride*, *Trichoderma citrinoviride*, *Trichoderma longibrachiatum*, *Verticillium intertextum*, *Acremonium* sp., *Acremonium strictum*, *Phaeoacremonium* sp., *Clonostachys rosea*, and *Scytalidium* sp. Among them, cytotoxic sorbicillinoids were isolated from the terrestrial fungus [201], and also from the marine-derived fungi *Trichoderma* sp., including bisorbicillinoids [157,158,159], *Penicillium terrestre* [160,161], *Penicillium* sp. [162], *Phialocephala* sp. [163,164,165,166], *Trichothecium* sp. [167], and *P. chrysogenum* [168]. All the above sorbicillinoid-producing species are from the Pezizomycotina subphylum from the Ascomycota phylum.

With interest in discovering new natural compounds with biological or therapeutic applications, researchers have also focused on studies of fungi of the *Trichoderma* genus to isolate new compounds. Rehman et al. [169] isolated and identified six sorbicillinoids (tricoreeseione A and B, tricodermolide B, 13-hydroxytricodermolide, 24-hydroxy-tricodimerol and 15-hydroxybisvertinol), and three analogs (trichodimerol, 24-hydroxy-bisvertinol, and bisvertinol) from *Trichoderma reesei* (HN-2016-018), derived from the fresh internal tissue of an unidentified sponge collected in China. Among them, 24-hydroxy-trichodimerol exhibited great potential for anticancer drugs, being cytotoxic against A549 lung carcinoma cell lines, MCF-7, and HCT116 cell lines with IC50 of 5.1, 9.5, and 13.7 μM, respectively [169]. Similarly, bislongiquinolide (trichotetronine) and dihydrotrichodimerol were isolated from *T. citrinoviride* and inhibited the proliferation of human cancer cell lines (U373 glioblastoma, Hs683 oligodendroglioma, A549, OE21 esophageal cancer, and SKMEL-28 melanoma), and B16F10 mouse melanoma cancer cell lines significantly through cytostatic and not cytotoxic activity [124]. Literature data show that dihydrotrichodimerol acts by activation of peroxisome proliferator-activated receptor-Υ (PPAR-Υ), which has an important role in cancer cell biology, being able to suppress the production of tumor necrosis factor-R (TNF-R) and nitric oxide in LPS-stimulated RAW264.7 cells [202].

The endophytes *F. oxysporum* CFE177 and *A. oryzae* CFE108 were isolated from the Brazilian medicinal plant *Combretum leprosum*, and their SMs present in the crude extract obtained from their culture showed cytotoxicity on the HeLa, ECV304, B16F10, J744, P388, Jurkat and k562 cell strains [203].

The *Cordyceps taii* species is normally found as a parasite in insect or arthropod larvae and produces the compounds deacetylcytophasin C and zygosporin D, which by the sulforhodamine B (SRB) method were cytotoxic for the 95-D human lung cancer cells. The cytotoxicity was more effective than that of the antitumor cisplatin [170].

Arora et al. [171] demonstrated that ylarone A and (-)5-methylmelein metabolites produced by the fungus *Xylaria psidii*, isolated from the leaf of the medicinal plant *A. marmelos*, have a cytotoxic effect against pancreatic cancer cells (MIA PaCa-2). These metabolites interrupt the cell cycle in the sub-G1 phase and induce cell death by activating apoptotic mechanisms.

The endophytic fungus *Diaporthe terebinthifolli*, isolated from the rhizome of *Glycyrrhiza glabra*, a native of India, produces bioactive compounds such as diapolic acid AB, xylarolide, and fomolide. These compounds were assayed for *in vitro* cytotoxicity using the cancer cell lines MIA PaCa-2 (pancreatic cancer), A549 (human lung adenocarcinoma), HCT-116 (human colon cancer), and T47D (human breast cancer). The xylarolide inhibited the growth of T47D cells with IC50 of 7 μM, through induction of apoptosis, and, in association with doxorubicin, inhibited the formation of tumor cell colonies [172].

In 2012, pestalrone B, an oxysporone derivative from the endophytic fungus *Pestalotiopsis karstenii* isolated from stems of *Camellia sasanqua*, showed significant cytotoxicity against the HeLa, HepG2, and U-251 cancer cell lines with IC50 of 12.6, 31.7, and 5.4 μg/mL, respectively [173]. In 2013, the compound siccayne [2-(3-methyl-3-buten-1-ynyl) hydro], a quinone isolated from the endophytic fungus *Pestalotiopsis fici* was cytotoxic against the human cancer cell lines HeLa and HT29, with IC50 of 48.2 and 33.9 μM, respectively [204].

Mady et al. [205] identified the molecules meleagrin, roquefortine C, and dehydro-histidyltryptophenyl-diketopiperazine (DHTD) produced by the endophytic fungus *P. chrysogenum* isolated from the leaf of the olive tree *Olea europea*. Roquefortine C and DHTD showed moderate antiproliferative activity against human breast cancer cells of the lines MDA-MB-231, MDA-468, BT-474, SK BR-3, MCF7, and MCF7-dox, while the compound meleagrin showed high inhibition in the growth of these same cell lines. In addition, the three compounds showed minimal toxicity to the MCF-10A non-tumorigenic human mammary epithelial cells [205], indicating its specificity for tumor cells.

A study published by Momesso et al. [174] showed that the fungus *Chaetomium globosum*, isolated from the leaves of *Viguiera robusta*, produced the chaetoglobosins B, D, and E. Chaetoglobosins are typically cytotoxic molecules, and the chaetoglobosin B derivative showed 89.55% and 57.10% inhibition of the growth of the tumor cells, Jurkat (leukemia) and B16F10 (melanoma), at 0.1 mg/mL.

Li et al. [175] also studied the cytotoxicity of SMs from the fungus *C. globosum*, isolated from *G. biloba*, and chaetoglobosins A, F, Fex, and 20-dihydrochaetoglobosin A showed IC50 values ranging from 3.15 to 8.44 μM on HCT116 cells, compared to the control drug, etoposide (IC50= 2.13 μM).

In a study performed by Hawas et al. [176], the fungus identified as *Penicillium aculeatum* was isolated from the marine red alga *Laurencia obtusa* and produced the sulfonyl metabolites pensulfonoxy and pensulfonamide. Preliminaries assays showed that these compounds were more cytotoxic than the antitumor paclitaxel. Pensulfonamide was cytotoxic to MCF-7 cells and pensulfonoxy to HCT-116 cells with IC50 of 2.18 and 5.23 µM, respectively.

*Penicillium polonicum* TY12 is a terrestrial endophytic fungus from the roots of *Aconitum vilmorinianum* from which a new indole-type monoterpene, polonidine A, and the already described compounds cyclopenol, verrucosidin, fructigenine A, 3-*O*-methylviridicatin, and aurantiomides C were isolated [177]. The cytotoxicity of these compounds was tested on the cell lines MHCC97H (human hepatoma), BT549 (human breast cancer), H1299 (lung cancer), SW620 (colon cancer), T98G (human glioma), and on the A549 (human lung cancer cell). Polonidine A exhibited moderate cytotoxicity on MHCC97H, BT549, and SW620 cell lines with IC50 from 6.1 to 20 µg/mL, while fructigenine A was less effective with IC50 of 20 µg/mL on T98G cells.

From the culture of the fungus *A. terreus* P63, endophyte of the grass *Axonopus leptostachyus*, was isolated the prenylated indole alkaloid, giluterrin, with an unprecedented carbon skeleton, which showed an antiproliferative effect on prostate (PC-3) and kidney (786-0) cancer cell lines [178].

### 6.2. Antimicrobial Compounds

The research for new antimicrobial molecules derivating from fungi metabolism is promising mainly due to the diversity of microorganisms not yet studied, which can contribute to the development of more specific drugs, with lower cost and effectiveness, even against drug-resistant pathogens.

Microbial resistance is considered a global health problem, which compromises the effectiveness of antibiotics, making the treatment of common infections unfeasible. Resistance occurs when microorganisms undergo genetic mutation due to intense exposure to antimicrobial drugs, and these microorganisms are considered superbugs. During the mutation phenomenon, bacteria, as well as fungi and yeasts, are able to develop strategies to prevent the antimicrobial effect of antibiotics, and are able to grow and multiplicate, promoting the emergence of drug-resistant strains.

Despite the severity of microbial resistance, the development of new antibiotics in the last decades has been rare. Although the incentives from the traditional market were not enough in the last years, it can hardly be able to solve the innovation gap in this area, especially in the context of restricted use of these drugs and sustainable discovery and development of new antibiotics [206]. Table 2 presents several examples of antimicrobial fungal SMs described in the last years.

**Figure 6:**
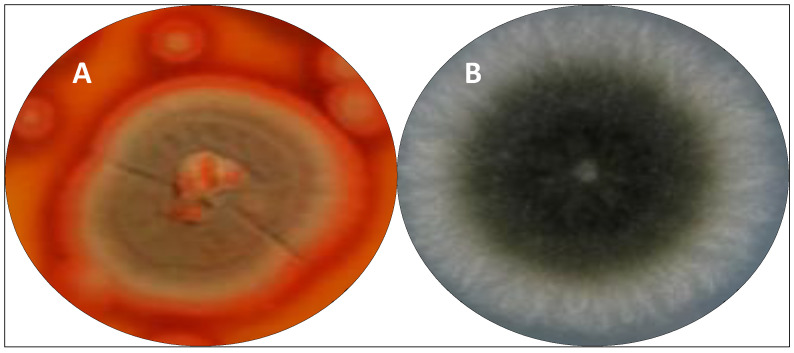
Illustration of the fungi species (**A**) *P. maximae* and (**B**) *E. rostratum* isolated by our group from the Brazilian biome, Caatinga.

Since the discovery of penicillin **(1)**, Penicillium species have been known and widely studied as important sources of new antimicrobial compounds. Among them, the *Penicillium* sp. F37 isolated from the marine sponge *Axinella corrugata* and closely related to *Penicillium maximae* (Figure 6) produced a novel chlorinated polyketide with 6,7-dihydro-4(5H)-benzofuranone moiety, which was characterized by spectroscopic methods as arvoredol [179]. This polyketide prevented biofilm formation by *Staphylococcus epidermidis*, and was also active in inhibiting the colorectal carcinoma HCT116 cells proliferation at 7.9 μg/mL.

The fungus identified as *P. aculeatum*, isolated from the marine red alga *L. obtuse*, is able to produce the sulfonyl metabolites pensulfonoxy and pensulfonamide. The crude ethyl acetate extract obtained from the culture product of this fungus inhibited the growth of *E. coli*, while the purified compound pensulfonamide inhibited the growth of *C. albicans* [176].

In another study with the fungus *P. chrysogenum* XNM-12 isolated from the seaweed *Leathesia nana*, the compounds, oxalicin C, penicierythritols A and B, and the monoterpenes decaturins B, C, D, and F were isolated [7]. The antimicrobial activity of the compounds was tested against *E. coli*, *Micrococcus luteus*, *P. aeruginosa* and on the phytopathogens *Ralstonia solanacearum*, *Valsa mali*, *A. alternata*, *Botrytis cinerea*, *F. oxysporum*, and *Penicillium digitatum*. Oxalicine C and penicierythritol A showed a moderate antibacterial effect on the bacterium *R. solanacearum* with MICs values of 8 and 4 μg/mL, respectively. Penicierythritol A possesses moderate antifungal properties against *A. alternata*, showing an MIC value of 8 μg/mL [7].

*Sophora tonkinensis* is a plant commonly applied in traditional Chinese medicine for the treatment of various diseases. From the roots of this plant, Qin et al. [208] isolated the fungus *Penicillium vulpinum*, which produced seven SMs derived from andrastin. These SMs were identified as 10-formyl andrastone A, 10-dymethylated andrastone A, 15-deacetylated citreohybridone E, citreohybridonol, and as andrastins A, B, and G. Except for andrastin G, the antibacterial activity was evaluated for all the others compounds that showed moderated effect with MIC ranging from 6.25 to 25 µg/mL on *Bacillus megaterium*, *Bacillus paratyphosus*, *Enterobacter aerogenes*, *E. coli*, and *S. aureus* [208].

Among the SMs obtained from the terrestrial fungus *P. polonicum* TY12, the compounds cyclopenol, verrucosidin, and fructigenine A showed a weak antibacterial effect with MIC of 643 µg/mL on *S. aureus* [177]. In spite of that, polonide A was the most effective, with MIC of 4 and 32 µg/mL on *S. aureus* and *B. subtilis*, respectively.

From the Brazilian biodiversity, the fungus *Exserohilum rostratum* (Figure 6) was isolated from *Bauhinia guianensis* [209] and *Phanera splendens* [210], which are typical Amazonian plants used in popular medicine to combat infections. Among the SMs produced by this species are the polyketide monocerin, ergosterol peroxide, annularins, and ravenelin, which presented moderated antimicrobial activity on *B. subtilis*, *S. aureus*, *E. coli*, *P. aeruginosa*, and *B. subtilis* and *Salmonella typhimurium* [209,210].

In 2018, from the endophytic fungus *Emericella* sp. XL 029, associated with the leaves of *Panax notoginseng*, four new polyketides (emericelactone A-D) were isolated and showed moderate antimicrobial activity against the phytopathogenic fungi *Verticillium dahliae Kleb, Rhizoctonia solani* and *Gibberella saubinetii*, and on the human pathogenic bacteria *Micrococcus lysodeikticus* and *Salmonella typhi* [211]. Emericelactone A, an optically active brown oil, contained on its structure an unprecedented linear pentaene substructure ending in an oxabicyclo[2.2.1]heptane moiety, while emericelactone B-D was characterized as epimers with an unprecedented linear triene structure with two cyclic moieties of an oxabicyclo[2.2.1]heptane and a cyclopentan-1-one. This study was the first one reporting the presence of emericelactones as SMs in Emericella species, suggesting that these polyketides might be acting as phytoalexins on the *P. notoginseng*, protecting it against pathogen attack. In the same year, another study with this same endophyte, demonstrated the isolation of two novel sesquiterpenoids, emericellins A and B, and both compounds showed moderate antimicrobial activity against *B. subtilis*, *Bacillus cereus*, and *E. coli* and on the fungi *V. dahliae* Kleb, *Helminthosporium maydis*, and *B. dothidea* [212].

The above-described compounds produced by *Alternaria* sp., alterperylenol, stemphyperylenol, 7-acetyl-1,3,6-trihydroxyanthracene-9,10-dione, (11*S*)-1,3,6-trihydroxy-7-(1-hydroxyethyl)anthracene-9,10-dione, and 7-acetyl-1,3,6-trihydroxyanthracene-9,10-dione showed an antimicrobial effect on phytopathogens [136]. Alterperylenol showed prominent antibacterial activity through membrane hyperpolarization without evidence of destruction of cell membrane integrity against *Clavibacter michiganensis* with MIC of 1.95 µg/mL, while the positive control streptomycin showed MIC of 3.90 µg/mL. 7-acetyl-1,3,6-trihydroxyanthracene-9,10-dione showed potent antibacterial activity toward the phytopathogenic bacteria *Pseudomonas syringae* pv. *lachrymans*, *Acidovorax avenae*, and *Erwinia carotovora* with MIC values of 3.91, 3.91, and 7.81 µg/mL, respectively, whereas (11*S*)-1,3,6-trihydroxy-7-(1-hydroxyethyl)anthracene-9,10-dione showed weak antibacterial effect against the same bacterial strains with MIC in the range of 7.81 to 15.6 µg/mL.

Alterperylenol showed antifungal activity with MIC values of 7.81 and 125 µg/mL against *Pestallozzia theae* and *Alternaria brassicicola*, respectively. 7-acetyl-1,3,6-trihydroxyanthracene-9,10-dione and (11*S*)-1,3,6-trihydroxy-7-(1-hydroxyethyl)anthracene-9,10-dione displayed moderate antifungal activity on *P. theae* with MIC value of 31.3 µg/mL (carbendazim MIC: 7.81 µg/mL) and stemphyperylenol displayed potent antifungal activity against *P. theae* and *A. brassicicola* with MIC values equal to those of carbendazim (carbendazim MIC: 7.81 μg/mL) [136].

Produced by the endophytic *B. dothidea* of *M. azedarach*, stemphyperylenol inhibited the phytopathogens *Alternaria solani* with MIC of 1.57 μM, being comparable to the commonly used fungicide carbendazim, and pycnophorin significantly inhibited the growth of *B. subtilis* and *S. aureus* with MIC of 25 μM [135].

The SMs engyodontiumones H, aspergillusone B, and AGI-B4 from *E. album* DFFSCS021 showed mild antibacterial activity against *E. coli* and *B. subtilis*, and aspergillusone B presented potent antilarval activity against barnacle *Balanus amphitrite* larval [183].

Four bioactive compounds were isolated from the fungus *Irpex lacteus* DR10-1; an endophytic from the water-tolerant plant *Distylium chinense*. The compounds were identified as the tremulane sesquiterpene irpexlacte A, and the furan derivatives irpexlacte B (5-(2α-hydroxypentyl) furan-2-carbaldehyde), irpexlacte C (5-(1α-hydroxypentyl) furan-2-carbaldehyde), and irpexlacte D (5-(5-(2-hydroxypropanoyl) furan-2-yl) pentan-2-one). These metabolites exhibited moderate antimicrobial activity against *P. aeruginosa* with MIC of 23.8 to 35.4 μM [213]. It is interesting that irpexlacte A and B presented remarkable antioxidant activity, and this effect may be related to the survival of the plant *D. chinense* under waterlogging stress, due to the release of ROS.

Although the Fusarium genus is known to harbor pathogenic species for plants, animals, and humans, several compounds isolated from species belonging to this genus are pointed out as antimicrobial agents [149,203,214,215,216,217,220,221]. In this regard, the fungus *F. oxysporum* CFE177, an endophyte from the Brazilian medicinal plant *C. leprosum*, produced SMs not yet identified, which showed an antifungal effect on important clinical species of C. *albicans*, *Candida krusei*, *Candida glabrata*, *Candida guillermondi*, *C. parapsilosis*, *C. tropicalis*, *Cryptococcus neoformans*, and on the filamentous fungi *Trichophyton rubrum* [203]. From this same plant, the endophyte *A. oryzae* CFE108 also produced antimicrobial SMs [203].

The SM fusarithioamide B was biosynthesized by the endophytic fungus *Fusarium chlamydosporium* isolated from the leaves of the plant *Anvillea garcinii* (Burm.f.) DC. On the bacteria *S. aureus*, *B. cereus*, and *E. coli*, the fusarithioamide B was more effective than the antimicrobial agent ciprofloxacin showing MIC from 2.5 to 3.7 µg/mL [215].

The cyclodepsipeptide, beauvericin isolated from *F. oxysporum* 5-19 endophytic on *Edgeworthia chrysantha* Linn showed an antimicrobial effect (MIC = 3.91 μM) on *S. aureus* similar to that of amoxicillin [217]. Furthermore, when produced by *F. oxysporum* EPH2R_AA_ and CEC1S, beauvericin exhibited antibacterial activity towards methicillin-resistant *S. aureus* (MIC = 3.125 μg/mL) and *Bacillus subtilis* (MIC = 3.125 μg/mL) [148].

The chemical investigation of SMs produced by *Fusarium solani* endophyte from *Casia alata* resulted in the isolation of the three naphthoquinones (anhydrofusarubin, fusarubin, and 3-deoxyfusarubin), one aza-anthraquinone (bostrycoidin), two sterols (ergosterol and 3,5,9-trihydroxyergosta-7,22-diene-6-one) and 4-hydroxybenzaldehyde. Beyond cytotoxicity, the napthoquinones and aza-anthraquinones exhibited antimicrobial and antioxidant effects; however, napthoquinone, aza-anthraquinone (bostrycoidin) was more bioactive [216]. Fusarubin was also found in *Cladosporium* sp. described as endophytes from the leaves of *Rauwolfia serpentina* [180]. Lately, the cytotoxicity of fusarubin and anhydrofusarubin was demonstrated through the inhibition of cell proliferation and an increase in apoptosis mediated by p21/p53- pathway [180].

The endophyte *F. oxysporum* ZZP-R1, from the coastal plant *Rumex madaio* Makino, used in traditional Chinese medicine, produced the two terpenes, fusariumins C and D, which showed an antibacterial effect on *S. aureus* with MIC of 6.25 µM and 25.0 µM [162]; however, both compounds were not effective on *E. coli* and *C. albicans*.

The 4-hydroxycinnamic acid derivative, methyl 2-{E-2-[4-(formyloxy)phenyl]ethenyl}-4-methyl-3-oxopentanoate, was isolated from the endophyte *Pyronema* sp. (A2-1 and D1-2) and expressed moderate antimycobacterial effect on *Mycobacterium marinum* [218].

The antimicrobial potential of the metabolites penixillarins A-C, and the biogenetically related 1,3-dihydroxy-5-(12-hydroxyheptadecyl)benzene and 1,3-dihydroxy-5-(12-sulfoxyheptadecyl)benzene obtained from the coculture of *P. crustosum* PRB-2 and *Xylaria* sp. HDN13-249A was evaluated on *Mycobacterium phlei*, *B. subtilis*, *Vibrio parahaemolyticus*, *E. coli*, *P. aeruginosa*, *C. albicans*, and *P. vulgaris* [207]. Among them, only the compounds 1,3-dihydroxy-5-(12-hydroxyheptadecyl)benzene, 1,3-dihydroxy-5-(12 sulfoxyheptadecyl)benzene, and penixillarins B and C were effective on *M. phlei*, *B. subtilis*, and *V. parahaemolyticus* with MIC in the range from 6.25 to 100 µM [207].

The ethyl acetate (EA) extract and the *p*-coumaric acid obtained from the endophytic fungus *A. alternata* isolated from *C. roseus* leaves showed concentration-dependent broad-spectrum antimicrobial activity against *E. coli*, *Klebsiella pneumoniae*, *P. vulgaris*, *P. aeruginosa*, *S. typhimurium*, *S. aureus*, and *Streptococcus faecalis*, and on the yeast *C. albicans* and *C. neoformans* [222]. These EA extracts and the *p*-coumaric acid were also effective as antifungal on the phytopathogens *A. brassicicola*, Alternaria geophila, A. flavus (aflatoxigenic strain), *A. fumigatus*, Aspergillus ochraceus, A. tamarii, A. terreus, Curvularia tetramera, F. oxysporum, Fusarium lateritium, F. equiseti, Fusarium udum, Fusarium verticillioides (fumonisinogenic strain), *P. citrinum*, and *Penicillium expansum*, showing MIC from 7.8 to 250 µg/mL. The authors suggested that the EA extract and *p*-coumaric acid could be alternatives to be explored as protection for preventing microbial deterioration and mycotoxins accumulation in food and feedstuffs storage.

In 2016, Shin et al. [219] performed a screening for the discovery and characterization of antimicrobial compounds from 342 entomopathogenic fungi strains in Korea, and showed that the obtained SMs completely suppressed the growth of *B. cereus* and *B. cinerea*. Although the compounds were not purified and physically chemically characterized, the results demonstrated that entomopathogenic fungi could be a promising source of antimicrobial compounds.

### 6.3. Cytotoxic and Antimicrobial Compounds Produced by Extremophile Fungi

Microorganisms that thrive under extremely adverse environmental conditions are classified as extremophiles [223], and possess mechanisms developed to adapt to these conditions through the regulation of the expression of specific genes in their genome. The extremophiles’ classification takes into account the environmental condition of the habitat in which they can grow and survive. The parameters for the classification are usually based on extremes of temperature (thermophiles, hyperthermophiles, psychrophiles), pH (acidophiles, alkaliphiles), pressure (piezophiles), salinity (halophiles), nutrient availability (oligotrophic), desiccation (xerophilesdry), metal concentration (metallophiles), radiation (radiophiles), and oxygen (microaerophiles). Moreover, there are polyextremophiles adapted to more than one extreme physicochemical factor.

The survival strategy of extremophiles can result in the biosynthesis of enzymes and SMs that can have applications in distinct areas such as environmental, industrial, and medical [224,225]. In light of that, extremophiles are considered an excellent source of new bioactive molecules [226]; however, currently, few biomolecules obtained from extremophiles have had successful biotechnological industrial applications. Besides that, due to several different reasons, extremophile microorganisms are not deeply studied concerning the chemistry of natural compounds and their potential as bioactive agents.

Literature data have shown that the majority of fungi species are able to tolerate extreme conditions of temperature, keeping the production of different thermostable enzymes, or SMs, useful in several distinct areas. Previously, the main enzymes and SMs produced by extremophile fungi were discussed by our group [227], including aspects related to mechanisms involved in their biosynthesis. Regarding this issue, Baranova et al. [228] summarized antibiotics described from 2018–2019, which resulted from the biosynthesis of extremophilic micromycetes, and classified these SMs according to the structure and biosynthetic origin.

In this review, Table 3 shows examples of cytotoxic and antimicrobial SMs produced by extremophilic fungi species. As an example, the species *Emericellopsis alkalina* is an extremophilic fungus found in waters with high levels of salinity and alkalinity, and these environmental conditions can contribute to the production of a great diversity of biological compounds, such as a peptaibol called emericellipsin A, which, unlike other peptaibols, has a smaller structure with nine residues [229]. Emericellipsin A showed cytotoxicity on the cell line HepG2 and HeLa *in vitro*, with IC50 of 2.8 and < 0.5 μM, respectively. In addition, this compound had less toxicity on fibroblast cells than the antitumor drug doxorubicin, showing its specificity for tumor cells.

In addition to previous studies [209,210], the fungus *E. rostratum* was isolated by our group from the endemic plant *Croton blanquetianus* from the Caatinga, a Brazilian biome with 844,000 km^2^, along with a semi-arid climate considered extreme, and its SMs, monocerin, and annularin I, showed moderated antimicrobial activity and absence of toxicity on human vein endothelial cells (HUVECs), inducting cell proliferation [unpublished data]. This species has intense melanin-based pigmentation, being resistant to stresses induced by UV and ionizing radiation, desiccation and heat, toxic metals, and organic pollutants [230].

A total of 52 fungal strains were isolated from Lake Magadi, in the East African Rift valley and phylogenetically classified as belonging to 18 different genera, represented mainly by Aspergillus, Penicillium, Cladosporium, Phoma, and Acremonium [231]. According to the conditions of growth (elevated temperature, pH, and salts), the isolates were of haloalkaliphilic nature, and different strains produced enzymes such as chitinases, proteases, amylases, cellulases, lipases, and pectinases. The isolate coded as 11M was identified as *P. chrysogenum* and produced antimicrobial SMs present in cell-free extracts and crude extracts which inhibited the growth of *B. subtilis*, *E. coli*, *P. aeruginosa*, *Salmonella* spp., *Shigella* spp., *C. albicans* and of the phytopathogens *Schizophyllum commune*, *Epicoccum sorghinum*, *A. fumigatus*, *Cladosporium halotolerans*, *Phoma destructive*, and *Didymella glomerata* [231]. The study was important to show that diversified culture conditions are an effective strategy to recover more phylotypes from extremes environment.

Three glacial ice fragments from the Antarctica Peninsula, of approximately 20 kg each, were fragmented in small pieces, melted and, after filtration of 3 L in a membrane of 0.45 µM, the retained material was inoculated in Sabouraud agar and minimal medium containing chloramphenicol and incubated at 10 °C for 60 days [232]. Following the process, 66 strains were isolated and classified into 27 taxa of 14 genera. The dominant species were *Penicillium palitans*, *Penicillium* sp., *Thelebolus balaustiformis*, *Glaciozyma antarctica*, *Rhodotorula mucilaginosa*, and *Rhodotorula dairenensis*. Crude extracts obtained from the culture of *P. chrysogenum*, *P. palitans*, and *Penicillium* spp. displayed leishmanicidal, trypanocidal, and herbicidal activities, with *P. chrysogenum* extract presenting the broadest and highest biological effect.

The strain of *P. chrysogenum* CCTCC M 2020019 was isolated from the feces of Adelie penguins in the Antarctic and its SMs, xanthocillins X and Y1, showed intense cytotoxicity against SF-268, MCF-7, HepG2 and A549 cancer cell lines with IC50 values ranging from 0.26 to 5.04 μM [233]. Xanthocillin X inhibited the growth of the Gram-negative pathogens *Acinetobacter baumannii*, *K. pneumoniae*, and *P. aeruginosa* growth with MIC values of 0.125 μg/mL [233]. Gubiani et al. [181] described that the soil-derived fungus *Aspergillus* sp SDC28, isolated from a Brazilian cave, produced the anthraquinones versicolorin C and versiconol, which were cytotoxic on human ovarian cancer cells (OVCAR3) with IC50 of 0.24 and of 1.07 μM, respectively, while the IC50 for taxol was 7 ± 2 nM. Beyond the cytotoxic effect, the study was the first one to report the spectroscopic characterization, including the absolute configuration of both anthraquinones, attesting to the relevance in drug discovery using extremophile fungi.

**Table 3:** Examples of cytotoxic and antimicrobial secondary metabolites produced by extremophilic fungi species.

Metabolites	Fungi Species	References
Xanthocillins X (cytotoxicity and antimicrobial) and Y1 (antimicrobial)	*P. chrysogenum* CCTCC M 2020019	[181]
Monocerin and annularin I (antimicrobial)	*E. rostratum*	[209,210]
Emericellipsin A (cytotoxicity)	*E. alkalina*	[229]
Versicolorin C and versiconol (cytotoxicity)	*Aspergillus* sp. SDC28	[234]

As a strategy to increase the production of bioactive compounds by different species of microorganisms, coculture was used for the culture of the deep-seawater derived fungus from Antarctica, *Penicillium crustosum* PRB-2, and of the mangrove fungus *Xylaria* sp. HDN13-249A [207]. Six SMs classified as polyketides were obtained and named penixillarins A-D, 1,3-dihydroxy-5-(12-hydroxyheptadecyl)benzene, and 1,3-dihydroxy-5-(12-sulfoxyheptadecyl) benzene. Through UPLC-MS analysis, it was shown that penixillarins A and B were produced by the interaction of both fungi species. The compounds penixillarin C and 1,3-dihydroxy-5-(12-sulfoxyheptadecyl)benzene were produced only by *Xylaria* sp. HDN13-249; however, in a much higher concentration under the cocultivation conditions. The cytotoxicity of all six metabolites was evaluated on 12 cell lines (HL-60, K562, BEL-7402, HCT-116, A549, HeLa, L-02, MGC-803, HO8910, SH-SY5Y, PC-3, and MDA-MB-231); nevertheless, no compound was effective until the concentration of 30 µM [207].

## 7. General Considerations

According to Wang et al. [234], from 1998 to 2019, 272 antimicrobial compounds were obtained from marine fungal strains isolated from sediments, sponges, algae, mangroves, and corals. China was the country responsible for the discovery of the majority of these compounds, which were obtained mainly from *Aspergillus* sp. followed by *Penicillium* sp. In spite of the fact that 207 compounds showed antibacterial activity and 68 were antifungal agents, none of them are in clinical trials.

In a wider period, between 1981 and 2019, among 164 new antimicrobial drugs approved, 36 were of biological origin, of which 89% were prophylactic agents. In the same period, although fungal diseases were frequently reported, only two antifungal drugs were approved, both of synthetic origin [1].

In addition to the indiscriminate use of antibiotics by the population around the world, microorganisms’ resistance to antimicrobials is also related to the high demand for animal protein for human consumption due to the regular use of antimicrobials in the modern production of this food. According to Van Boeckel et al. [235], the global average consumption of antimicrobials is from 45 to 172 mg/kg of animal produced per year, considering cattle, chicken, and pigs. In this sector, globally, the use of antimicrobials tends to increase by 67% in developed countries between 2010 and 2030, while in countries with low *Human Development Index* (HDI), such as Brazil, Russia, India, China, and South Africa, this rate may increase, reaching 99% [235].

Historically, it is clear that drug discovery is still not enough to attend to the high demand for new antimicrobial and antitumor agents, evidencing the relevance of intensifying studies in order to have a sustainable use of the huge world biodiversity, as well as of microorganisms.

Although the production of SMs by fungi is not always economically interesting due to the low yield from the fermentation process, potential species can be selected and genetically engineered to enhance the production of specific bioactive compounds through sustainable processes. Furthermore, chemical processes can be developed by medicinal chemistry for the production of such compounds with high yield, or their structure can be modified to reach specific antitumor effects.

## 8. Conclusions

The studies involving fungal SMs have been increased due to their relevance to biotechnology, medicine, and the pharmaceutical industry. Several SMs possess different pharmacological properties, and in an effort to discover anticancer and antimicrobial compounds and to attend to the high demand, research groups from all around the world are intensely exploring this possibility. In this review, various molecules from different chemical classes produced by fungi metabolism were presented, as well as their antimicrobial or cytotoxic effect. The SMs can be useful to improve the current treatments of infections and cancer, and their effects can go far beyond than described in this review. Some reported SMs are from endophytes isolated mainly from medicinal plants, which, in addition to their inherent effect, are also an interesting source of microorganism species and, consequently, from new molecules. Several SMs were assayed only *in vitro*, and their effects were not promising to encourage new assays; however, even these data are important for future screening and studies.

The relevance of fungi as a source of new molecules for the drug discovery process is evident; however, it is important to explore the compounds already described for obtaining new derivatives or analogs that can be more effective. As was discussed, fungi species producers of bioactive and promising SMs can also be engineered through molecular-based approaches and tools to increase their production, reduce adverse effects or improve their pharmacological properties. The main purpose is obtaining specific treatments with reduced or absence of severe side effects and low cost. Considering the wide and rich biodiversity around the entire world and all the available technology and knowledge accumulated over the years, this is a viable approach able to improve the quality of human life. 

## Figures and Tables

**Table 2:** Examples of antimicrobial secondary metabolites produced by fungi.

Metabolites	Fungi Species	References
Oxalicine C, penicierythritol A	*P. chrysogenum* XNM-12	[7]
Stemphyperylenol	*B. dothidea*	[135]
Alterperylenol, stemphyperylenol, 7-acetyl-1,3,6-trihydroxyanthracene-9,10-dione, (11*S*)-1,3,6-trihydroxy-7-(1-hydroxyethyl)anthracene-9,10-dione, and 7-acetyl-1,3,6-trihydroxyanthracene-9,10-dione	*Alternaria* sp.	[136]
Pensulfonoxy, pensulfonamide	*P. aculeatum*	[176]
Cyclopenol, verrucosidin and fructigenine A, polonide A	*P. polonicum* TY12	[177]
Arvoredol	*Penicillium* sp. F37	[179]
Fusarubin	*Cladosporium* sp.	[180]
Engyodontiumones H, aspergillusone B, AGI-B4	*E. album* DFFSCS021	[183]
1,3-dihydroxy-5-(12-hydroxyheptadecyl)benzene, 1,3-dihydroxy-5-(12 sulfoxyheptadecyl)benzene, and penixillarins B and C	Coculture of *P. crustosum* PRB-2 and *Xylaria* sp. HDN13-249A	[207]
10-formyl andrastone A, 10-dymethylated andrastone A, 15-deacetylated citreohybridone E, citreohybridonol, andrastins A and B	*P. vulpinum*	[208]
Ergosterol peroxide, monocerin, annularin I and J, ravenelin	*E. rostratum*	[209,210]
Emericelactone A-D, emericellins A and B	*Emericella* sp. XL 029	[211,212]
Irpexlacte A, furan derivatives irpexlacte B (5-(2α-hydroxypentyl) furan-2-carbaldehyde), irpexlacte C (5-(1α-hydroxypentyl) furan-2-carbaldehyde), irpexlacte D (5-(5-(2-hydroxypropanoyl) furan-2-yl) pentan-2-one)	*I. lacteus* DR10-1	[213]
Fusariumins C and D	*F. oxysporum* ZZP-R1	[214]
Fusarithioamide B	*F. chlamydosporium*	[215]
Anhydrofusarubin, fusarubin, 3-deoxyfusarubin, bostrycoidin, ergosterol, 3,5,9-trihydroxyergosta-7,22-diene-6-one, 4-hydroxybenzaldehyde	*F. solani*	[216]
Beauvericin	*F. oxysporum* EPH2R_AA_ and CEC1S *F. oxysporum* 5-19	[148,217]
4-hydroxycinnamic acid derivative, methyl 2-{(E)-2-[4-(formyloxy)phenyl]ethenyl}-4-methyl-3-oxopentanoate	*Pyronema* sp. A2-1 *Pyronema* sp. D1-2	[218]
*p*-Coumaric acid	*A. alternata*	[219]
